# Zone-MPC Automated Insulin Delivery Algorithm Tuned for Pregnancy Complicated by Type 1 Diabetes

**DOI:** 10.3389/fendo.2021.768639

**Published:** 2022-03-22

**Authors:** Basak Ozaslan, Sunil Deshpande, Francis J. Doyle, Eyal Dassau

**Affiliations:** Harvard John A. Paulson School of Engineering and Applied Sciences, Harvard University, Boston, MA, United States

**Keywords:** type 1 diabetes, model predictive control, automated insulin delivery, pregnancy, *in-silico* verification

## Abstract

Type 1 diabetes (T1D) increases the risk for pregnancy complications. Increased time in the pregnancy glucose target range (63-140 mg/dL as suggested by clinical guidelines) is associated with improved pregnancy outcomes that underscores the need for tight glycemic control. While closed-loop control is highly effective in regulating blood glucose levels in individuals with T1D, its use during pregnancy requires adjustments to meet the tight glycemic control and changing insulin requirements with advancing gestation. In this paper, we tailor a zone model predictive controller (zone-MPC), an optimization-based control strategy that uses model predictions, for use during pregnancy and verify its robustness *in-silico* through a broad range of scenarios. We customize the existing zone-MPC to satisfy pregnancy-specific glucose control objectives by having (i) lower target glycemic zones (i.e., 80-110 mg/dL daytime and 80-100 mg/dL overnight), (ii) more assertive correction bolus for hyperglycemia, and (iii) a control strategy that results in more aggressive postprandial insulin delivery to keep glucose within the target zone. The emphasis is on leveraging the flexible design of zone-MPC to obtain a controller that satisfies glycemic outcomes recommended for pregnancy based on clinical insight. To verify this pregnancy-specific zone-MPC design, we use the UVA/Padova simulator and conduct *in-silico* experiments on 10 subjects over 13 scenarios ranging from scenarios with ideal metabolic and treatment parameters for pregnancy to extreme scenarios with such parameters that are highly deviant from the ideal. All scenarios had three meals per day and each meal had 40 grams of carbohydrates. Across 13 scenarios, pregnancy-specific zone-MPC led to a 10.3 ± 5.3% increase in the time in pregnancy target range (baseline zone-MPC: 70.6 ± 15.0%, pregnancy-specific zone-MPC: 80.8 ± 11.3%, *p* < 0.001) and a 10.7 ± 4.8% reduction in the time above the target range (baseline zone-MPC: 29.0 ± 15.4%, pregnancy-specific zone-MPC: 18.3 ± 12.0, *p* < 0.001). There was no significant difference in the time below range between the controllers (baseline zone-MPC: 0.5 ± 1.2%, pregnancy-specific zone-MPC: 3.5 ± 1.9%, *p* = 0.1). The extensive simulation results show improved performance in the pregnancy target range with pregnancy-specific zone MPC, suggest robustness of the zone-MPC in tight glucose control scenarios, and emphasize the need for customized glucose control systems for pregnancy.

## 1 Introduction

Type 1 diabetes (T1D) is characterized by destruction of pancreatic *β*-cells and consequent lack of adequate endogenous insulin production to regulate blood glucose, for which values outside of a certain range may lead to health complications (1). Daily treatment of T1D aims to keep glucose values within a euglycemic range *via* external insulin delivery. In recent years, diabetes technologies have made great strides in providing more advanced devices that facilitate daily treatment to the extent that closed-loop control (CLC) has become an available treatment option. A CLC system, also known as artificial pancreas or automated insulin delivery, consists of a continuous glucose monitor (CGM) that periodically measures the subcutaneous glucose level, a computing unit that decides the amount of insulin to be injected, and a continuous subcutaneous insulin injection (CSII) pump that delivers the insulin to the person with T1D. CLC decreases the risk of both hypoglycemia and hyperglycemia, increases the time spent in the clinically recommended glucose range (70-180 mg/dL) (2, 3), and improves the quality of daily life (4). While CLC systems are of great value for all individuals living with T1D, the currently approved ones are mainly designed for adults. Only a few have been approved for use in younger populations^1^^,^ ^2^, and one system that was developed in the U.K (5) bears CE marking for use of the system in people aged ≥ 1 year and also in pregnancy (6). However, there are no Food and Drug Administration (FDA) approved CLC system for use during pregnancy and the need for customization of these systems for different glucose control requirements remains largely unmet (7).

Pregnancy with T1D is one of the conditions that necessitate customization of CLC systems due to significant changes in the glucose-insulin metabolism with advancing gestation (8) and tight glucose control targets throughout gestation (9). The tight glucose targets are motivated by the association between poor glycemic control and higher risks of maternal, fetal, and neonatal complications (10). Therefore, treatment strategies that are tailored to the insulin and glucose control needs of pregnancy are imperative to improve pregnancy outcomes. Additionally, the role of CLC systems in mitigating the shortcomings of open-loop treatment is further emphasized during pregnancy since optimal adjustment of insulin treatment parameters across pregnancy stages is particularly difficult due to limited knowledge on the magnitude of metabolic changes and their variations across individuals.

The first CLC system tested during pregnancy complicated by T1D was in the U.K. (5, 11, 12). These trials leveraged the adjustable glucose target feature of an existing glucose controller for T1D to achieve pregnancy-specific targets (5, 13). The results were promising as the CLC improved glucose control over routine open-loop control methods. In this paper, we seek a solution to the following problem:

*Customize a T1D closed-loop glucose controller for use during pregnancy such that the resulting glucose profiles satisfy the clinical requirements across a comprehensive set of scenarios under which the system may operate.*

Zone-MPC is an optimization-based algorithm that uses model predictions to optimize insulin injections in a way that blood glucose levels are kept within a target zone (14–17). The zone objective differs from a specific set-point since all the glucose values within the zone are treated equally acceptable. Zone-MPC has proved safe and effective in improving glucose control in non-pregnant individuals with T1D (18–21). Building on this validated CLC architecture, the primary contributions of this work are:

- A CLC design based on zone-MPC that is tuned to the needs of pregnant women with T1D. We show that this is achieved by modifying certain parameters in the zone-MPC cost function and meal and correction bolus strategy.
- A robustness verification through *in-silico* experiments under a broad range of clinically possible scenarios. We present extensive numerical results outlining the performance trends for multiple parameters. We show that, for pregnancy targets, our design consistently outperforms the existing zone-MPC design as well as a *zone-adjusted* one, for which the details are made clear in the paper.

In the rest of the manuscript, we first provide the necessary background on the simulation environment, the zone-MPC algorithm, and clinical glucose control requirements during pregnancy complicated by T1D. Then, we formulate the problem of tuning and customization of zone-MPC for pregnancy. As a solution to the described problem, we provide the pregnancy-specific zone-MPC parameters and then, the results from the *in-silico* experiments. We also compare the performance of pregnancy-specific zone-MPC with the baseline zone-MPC as well the zone-adjusted MPC. Finally, we conclude the manuscript with discussions, the results from the controller’s validation in real-life, and an overview of future directions.

## 2 Methods

**Figure 1:**
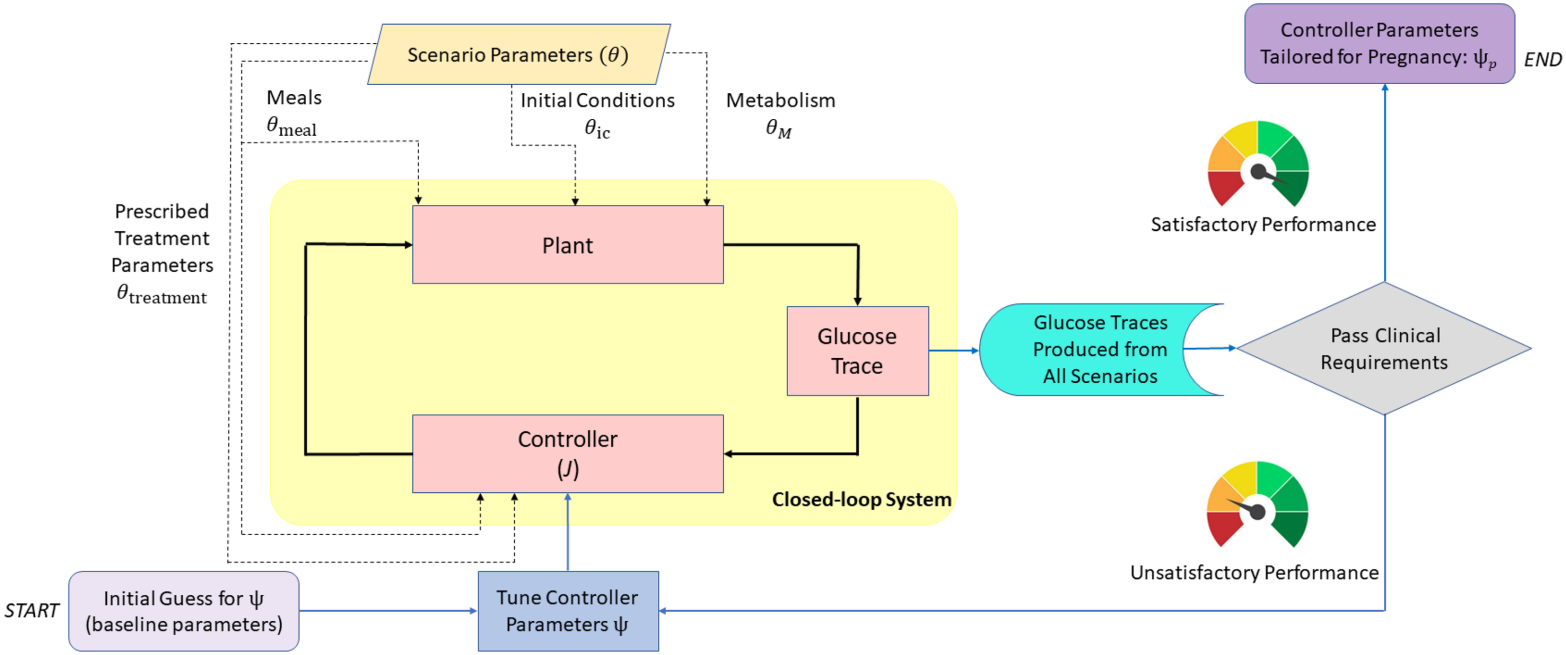
Block diagram describing the design and verification process of the zone-MPC controller for use in pregnancy. Closed-loop system is at the core (yellow shaded area). Scenario parameters (dashed lines) are fed into the simulator and the controller. The resulting glucose trajectories are evaluated for safety and performance. Controller parameters are tuned iteratively to obtain glucose outputs that satisfy the clinical requirements. Blue lines belong to the flow chart of this decision process. Note that the controller injects an optimal insulin input that minimizes the cost, *J*, at every time step.

A schematic overview of our approach is illustrated in **Figure 1**. The behavior of zone-MPC is determined by multiple parameters, denoted by *ψ*, for which the details are presented in the following sections. We tune *ψ* to pregnancy-specific glucose control requirements and also formalize a set of scenarios, parameterized with notation *θ*, under which the *in-silico* experiments are conducted. These scenarios capture challenging conditions such as variations in insulin sensitivity, which are known to take place during pregnancy. Our tuning problem can be viewed as finding *ψ* such that the robust performance is achieved for a chosen set of *θ*. This problem is formalized in *Section 2.7.1* after providing the necessary background in the leading sections.

### 2.1 Metabolic Model (Plant)

The underlying metabolism of glucose regulation has complex dynamics. In general, an insulin-meal-glucose model can be expressed in the following ordinary differential equation format:


(1) $$\frac{ds}{dt}=F(s,u_{\text{ins}},u_{\mathrm{glucose}};\theta_{M}),$$

where *s* is the metabolic state, *u*_ins_ ∈ ℝ_+_, is the insulin input, *u*_glucose_ ∈ ℝ_+_ is the external glucose input, and *θ_M_* is the set of patient-specific metabolic parameters. The input *u*_ins_ is determined by the insulin therapy method (e.g., multiple daily injections, insulin pump therapy, closed-loop insulin therapy), and *u*_glucose_ can be both in the form of intravenous injection or oral meal intake. Several mathematical forms for the function *F* in (1) have been developed in the literature. They range from minimal models such as the one in (22) to more comprehensive ones that include details such as oral glucose intake, hepatic glucose production, and glucose utilization as in (23, 24).

Our method uses the comprehensive model that is described in (24), which is at the core of the UVA/Padova S2013 simulator. This simulator is accepted by the FDA as a substitute for animal trials in testing safety and feasibility of new glucose control algorithms before human clinical trials. It is equipped with *in-silico* subjects for the three main age-groups (i.e., children, adolescents, and adults). In order to mimic pregnancy-induced changes to insulin-glucose metabolism of an *in-silico* adult cohort, we adjust *k_p_*_3_, a rate that affects insulin action in the liver denoted by *X^L^*, and *V_mx_*, a parameter that acts on insulin-dependent glucose utilization, in the following equations:


(2) $$EGP(t)=k_{p1}-k_{p2}G_{p}(t)-k_{p3}X^{L}(t)+\xi X^{H}(t)$$

(3) $$U_{id}(t)=\frac{\left[V_{m0}+V_{mx}X(t)(1+r_{1}risk)]G_{t}(t\right)}{K_{m0}+G_{t}(t)}$$

where *EGP* is the endogenous glucose production, *X^H^* is delayed glucagon action, *ξ* is liver responsivity to glucagon, *G_p_* is the amount of glucose in plasma, *G_t_* is the amount of glucose in the tissue, *U_id_* is insulin-dependent glucose utilization, *X* is insulin action on glucose utilization, *k_p_*_1_, *k_p_*_2_, *k_p_*_3_, *V_m_*_0_, *V_mx_*, *K_m_*_0_, and *r*_1_ are model parameters. Glycemic risk, denoted as *risk*, is a quantitative indicator of clinical risk associated with glucose values (25) and here, it is employed in the modeling of insulin-dependent glucose utilization. The full metabolic model and details can be found in (24).

The first trimester of pregnancy is characterized by a lower insulin requirement and the insulin requirements increase from mid-gestation onward (8). We vary *k_p_*_3_ and *V_mx_* in the experiments to evaluate the performance of different controller designs across a range of insulin sensitivity changes.

### 2.2 Periodic Zone Model Predictive Control (Zone-MPC)

In this section, we provide the necessary background on Zone-MPC, which is the feedback control component of glucose management in our framework. We present the mathematical details of the parts of zone-MPC that we tailor for use during pregnancy. Other details are presented at a higher level and the interested reader is referred to (15, 16), and (17) for further technical information, and to (18–21) for previous clinical trials that demonstrated safety and efficacy of the zone-MPC algorithm in adults and adolescents with T1D.

#### 2.2.1 Prediction Model

While metabolic models in the form of (1) are useful for high fidelity simulations, they are too complex for model-based controller design. Therefore, simpler models are used for computing insulin injection decisions in real time. We use the discrete-time model in (15) with the following state-space form:


(4a) x(k+1)=Ax(k)+Bu(k)

(4b) y(k)=Cx(k)

where:

• *x*(*k*) ∈ ℝ^3^ is the system state representing the three step differential blood glucose values at time *k* ∈ ℕ relative to a reference blood glucose value, denoted as *G_ref_*,


(5) $$x(k)=[G(k+2)-G_{ref,}G(k+1)-G_{ref},G(k)-G_{ref}]$$

• *u*(*k*) ∈ ℝ is the control input (insulin) at time *k* relative to the user’s pre-programmed time-dependent basal profile (U/hour), *β*(*k*):


(6) $$u(k)=u_{ins}(k)-u_{basal}(k)$$

(7) $$u_{basal}(k)=\beta (k)\frac{T_{s}}{60}$$

where *T_s_* denotes the time interval between *k* and *k* + 1 and is chosen as 5 minutes in accordance with the sampling period of current continuous glucose sensing technology.

• *y*(*k*) ∈ ℝ is the measured blood glucose level relative to *G_ref_*.

By employing a standard state observer (26), we obtain an estimate of *x* denoted by $\hat{x}$ , as follows:


(8) $$\hat{x}(k+1)=A\hat{x}(k)+Bu(k)+L(y(k)-C\hat{x}(k))$$

where *L* is the observer gain.

Note that (4) does not include *u*_glucose_ and the meal control is handled separately as described in *Section 2.3*. The numerical values for *A*, *B*, *C*, and *L* are available in the original source (15–17).

#### 2.2.2 Optimization Problem and Algorithm

The zone-MPC, similar to other MPC algorithms in control theory (27), uses mathematical optimization to make control decisions, and can accommodate constraints such as those on blood glucose and insulin (e.g., the amount of injected insulin can not be a negative number). At time *k*, we optimize *u*(0|k), u(1|k),… , *u*(*N_u_* - 1|*k*), where *u*(*j*|*k*) is the tentatively planned differential control input at time *k* + *j* relative to the prescribed basal at the same time, and *N_u_* is the control horizon. The mathematical optimization is written as:


(9) $$\begin{array}{l} u^{*}(0|k),u^{*}(1|k),\cdots ,u^{*}(N_{u}-1|k)=\underset{u(0|k),u(1|k),\cdots ,u(N_{u}-1|k)}{\text{argmin}}J\\ \begin{array}{cccccccccc} & & & & & & & & \text{subject}\;\text{to} & x(i+1|k)=Ax(i|k)+Bu(i|k), \end{array}\\ \begin{array}{cccccccccccccc} & & & & & & & & & & & & & x(0|k)=\hat{x}(k), \end{array}\\ \begin{array}{cccccccccccccc} & & & & & & & & & & & & & i=0,1,\cdots ,N_{p}-1, \end{array}\\ \begin{array}{cccccccccccccc} & & & & & & & & & & & & & u(k+j)+u_{\text{basal}}(k+j)\geq 0, \end{array}\\ \begin{array}{cccccccccccccc} & & & & & & & & & & & & & u(k+j)+u_{\text{basal}}(k+j)\leq u_{\text{UB}}(\tau (k+j)), \end{array}\\ \begin{array}{cccccccccccccc} & & & & & & & & & & & & & u(k+j)+u_{\text{basal}}(k+j)\leq u_{\text{IOB}}(\tau (k)), \end{array}\\ \begin{array}{cccccccccccccc} & & & & & & & & & & & & & u(N_{u})=u(N_{u}+1)=\cdots =u(N_{p}-1)=0, \end{array}\\ \begin{array}{cccccccccccccc} & & & & & & & & & & & & & j=0,1,\cdots ,N_{u}-1, \end{array} \end{array}$$

where *J* is the cost function that is described in *Section 2.2.3*, *τ*(∙) is the time of day in minutes after midnight corresponding to the discrete time-step index, *u_UB_* is the time-dependent insulin delivery upper bound, and *u_IOB_* is the insulin on board (IOB) dependent insulin delivery upper bound. The details of the insulin input constraints are provided in *Section 2.2.4*. By solving (9), the optimal plan u* (0|*k*),…, u* (*N_u_* – 1|*k*) is obtained. Only u*(0|*k*) is used to decide the insulin injection:


(10) $$u_{Zone-\text{MPC}}(k)=u^{*}(0|k)+u_{\text{basal}}(k).$$

Given the subsequent measurement *y*(*k*), the estimate of state $\hat{x}\left(k+1\right)$ becomes available and a new optimization problem is formed to find *u*_Zone – MPC_(*k *+ 1). In our implementation, the control horizon is 25 minutes and the prediction horizon is 45 minutes, represented by *N_u_* = 5 and *N_p_* = 9, respectively.

#### 2.2.3 Cost Function

Zone-MPC uses a zone-based cost function in the following form:


(11) $$J:=J_{1}+J_{2}+J_{3}$$

where the terms and their interpretations are given as:


(12a) $$J_{1}:=\sum_{j=1}^{N_{p}}(\|G_{ZL}(k+j)-G(k+j){\|}_{\gamma }^{2}+Q(v(k+j),\text{IOB}(k))\|G(k+j)-G_{ZH}(k+j){\|}_{\gamma }^{2}$$

(12b) $$J_{2}:=\sum_{j=1}^{N_{p}}\hat{D}(G(k))\|\frac{1}{10}(G(k+j)-G(k+j-2)){\|}_{y}^{2}$$

(12c) $$J_{3}:=\sum_{j=0}^{N_{u}-1}\left(R_{+}||u(k+j)|{|}_{\gamma }^{2}+R\_||-u(k+j)|{|}_{\gamma }^{2}\right),$$

and ||∙||*_γ_* notation is used for the following semi-norm:


(13) $$||\cdot |{|}_{\gamma }:=\max(0,\cdot ).$$

• The term *J*_1_ penalizes the deviation in the glucose levels outside the zone [*G_ZL_*, *G_ZH_*], where *G*_ZL_ and *G*_ZH_ are the lower and upper bounds of the control zone, respectively. The values of these bounds can be assigned in a time-varying manner as shown in (16). Note that ||*G_ZL_* – *G*||*_γ_* = ||*G* – *G_ZH_* ||*_γ_* = 0 if and only if G ∈ [*G_ZL_*, *G_ZH_*]. Also note that the penalization of above the zone glucose levels is weighed by the function *Q* that factors in the glucose velocity $v\left(k\right):=\frac{1}{10}\left[G\left(k+2\right)-G\left(k\right)\right]$ , and the amount of active insulin in the body, namely IOB. The function *Q* associates a higher cost to persistent hyperglycemia without a steep increase in glucose. Please note that this aspect of the controller is kept the same across controllers presented in this manuscript.

• The second term *J*_2_ penalizes the glucose velocity, defined as glucose level change over two time steps (2 × 5 minutes = 10 minutes). Velocity term was added to the later version of zone-MPC in (17) for better postprandial glucose management when the glucose response to meal poses high risk for hyperglycemia. The function $\hat{D}$ is defined as:


(14) $$\hat{D}(G)=\{{}_{1}^{D}{\;}_{otherwis\mathrm{e,}}^{G_{\_}^{v}\leq G\leq G_{+}^{v},}$$

where for *D* > 1, increasing glucose is penalized more heavily when glucose levels are within $\left[G_{\_}^{v},G_{+}^{v}\right]$ . The upper bound $G_{+}^{v}$ deactivates high velocity penalties since *J*_1_ already introduces high cost in the case of significant deviation from the zone.

• The last term *J*_3_ penalizes deviations of the candidate control action from the referenced basal profile *u*_basal_. Penalty on insulin inputs higher than *u*_basal_ are weighed by *R*_+_ and inputs lower than the reference are weighted by *R*_–_. Typically *R*_+_ is chosen higher than *R*_–_ compatible with the higher clinical risk associated with the hypoglycemia compared to the hyperglycemia as explored in (16). In this work, we have *R*_+_ and *R_–_* set to 6500 and 100, respectively.

#### 2.2.4 Insulin Input Constraints

The controller can suspend insulin injection based on the optimal insulin delivery plan obtained. However, the maximum insulin amount that can be injected is subject to the following constraints:


(15) $$u_{\text{UB}}(k):=\{\begin{array}{ll} 4u_{\text{basal}}(k), & T_{1}<\tau (k)<T_{2},\\ 1, & \text{otherwise}. \end{array}$$

(16) $$u_{\text{IOB}}(k):=\{\begin{array}{ll} \text{IO}{\text{B}}_{\text{required}}(k)-\text{IOB}(k)+u_{\text{basal}}(k), & \text{IO}{\text{B}}_{\text{required}}(k)>IOB(k),\\ u_{\text{basal}}(k), & \text{otherwise}. \end{array}$$

(17) $$\text{IO}{\text{B}}_{\text{required}}(k):=\frac{G(k)-G_{\text{ref}}}{CF},$$

where *T*_1_ is the start of daytime, *T*_2_ is the end of daytime, *CF* is the correction factor, and *IOB*_required_ is the estimated insulin required to bring the glucose level to *G_ref_*.

The IOB is calculated based on the history of insulin injection and an insulin decay curve where we can specify the length of insulin action to be used in the calculation. We denote the length of the IOB curves used to compute the IOB from the user-requested boluses and controller-delivered micro-boluses by *λ* and *μ*, respectively. While *λ* is a fixed parameter, *μ* is a function of the glucose level at the time of the calculation. The total IOB is calculated as a sum of the IOB from the user-requested boluses and the IOB from the controller-delivered boluses relative to the basal profile. Note that this aspect is not modified for this work. The full details are provided in (28), and Appendix A4 of (17). The shape of the IOB curves used in this work are illustrated in the **Supplementary Material**.

### 2.3 Meal and Correction Bolus Strategy

Meal bolus is applied in a feed-forward manner upon meal announcement by the user and computed as follows:


(18a) $$u_{\text{meal}}:=\{\begin{array}{cc} \frac{M}{CR_{i}}, & G>G^{m},\\ \frac{\alpha }{CR_{i}}, & \text{otherwise}, \end{array}$$

(18b) $$u_{\text{correction}}:=\{\begin{array}{cc} \text{min}(2,\frac{{\tilde{y}}_{i}-G_{\text{ref}}^{\text{corr}}}{CF_{i}}), & {\tilde{y}}_{i}\geq \tau_{c},\Delta_{i}>120,\\ 0, & \text{otherwise}. \end{array}$$

where *i* denotes the meal intake time, *M* is the carbohydrate amount in the meal expressed in grams, *CR_i_* is the subject and time specific carbohydrate ratio at the time of meal intake, *G^m^* is the glucose threshold for full meal bolus to be applied, and *α* is the bolus reduction factor when the glucose at the meal time is less than the full meal bolus threshold. A correction bolus, *u*_correction_ is added to the meal bolus based on the glucose level at the time of calculation, ${\tilde{y}}_{i}$ , a reference fasting glucose value, $G_{\text{ref}}^{\text{corr}}$ , a subject and time specific correction factor, *CF_i_*, glucose threshold for additional correction, *τ_c_*. In order for a correction bolus to be added, the time passed since the last correction bolus, denoted by Δ*_i_*, needs to be more than the minimum duration between two user-requested boluses, which is set as 120 minutes.

### 2.4 Clinical Glucose Control Requirements in Pregnancy Complicated by T1D

There are differences in the recommended glycemic targets for pregnancy compared to the general population with T1D. The target glucose range for pregnancy is 63 – 140 mg/dL which is tighter than the non-pregnancy target of 70 - 180 mg/dL. The consensus recommendation is to have less than 25% of the time above the target range, more than 70% time in the range, less than 4% of the time below 63 mg/dL in a way that time below 54 mg/dL does not exceed 1% (9). These consensus targets constitute the primary endpoint, in other terms success criteria, for the verification of the pregnancy-specific zone-MPC design. Of note, having a glucose control of <80-90 mg/dL is not recommended as too tight control has been associated with increased risk of limiting fetal development (29). There are also fasting and postprandial glucose control related targets: (i) fasting plasma glucose levels, measured after ≥ 8 hours of fasting or overnight fasting, are aimed to be below 95 mg/dL; (ii) *either* one-hour postprandial glucose should be below 140 mg/dL *or* two-hour postprandial glucose should be below 120 mg/dL (30). The postprandial glucose targets are specified for self-monitored glucose measurements since many CGM devices do not yet have an indication for pregnancy. However, in this work, we have the simulated CGM measurements and access to the full postprandial glucose excursions. Therefore, we evaluate the early postprandial control performance *via* the CGM time in the target range within two-hour following meal intake. The fasting and postprandial glucose control performances are secondary endpoints in our verification process.

### 2.5 Simulation Parameters

Here we describe how to setup the simulations. We can classify the parameters of interest for our work into two groups: (i) *scenario parameters*, which we denote by *θ*, and (ii) *controller parameters*, denoted by *ψ*. Given *ψ* and *θ*, one can simulate (1) and obtain a glucose trajectory: 


(19) G(ψ,θ)=G(0),G(1),⋯

Closed-form expressions for G(*ψ*, *θ*) are not available and we can only numerically compute G(*ψ*, *θ*). The tuning for robust performance demands that *θ* and *ψ* are designed *against each other* in a sense that is made clear in the subsequent sections.

#### 2.5.1 Scenario Parameters

First, we introduce the following notion of *scenario parameters* in formalizing our tuning problem:

- *θ_M_*: metabolic parameters of the subject with T1D. We employ 10 adult subjects in the UVA/Padova simulator and each subject has their own metabolic parameters. By varying these parameters, we can simulate outcomes for differing metabolic states (e.g., increased or decreased insulin sensitivity).
- *θ*_ic_: initial conditions of the simulation that is equivalent to *s*(0) in (1). By varying *θ*_ic_, one can test the system under a range of initial states, such as very high or very low initial glucose levels (i.e., *G*(0) far from the [*G*_ZL_, *G*_ZH_] zone).
- *θ*_treatment_: insulin treatment parameters (i.e., *β*, *CR*, *CF*) that are typically prescribed by the healthcare provider. By varying *θ*_treatment_, we explore scenarios where subjects’ treatment parameters are properly adjusted for pregnancy, or are not properly adjusted and need to be compensated by the controller.
- *θ*_meal_: parameters characterizing the time and size of the carbohydrates consumed, and the timing of meal boluses relative to the meal intake time. Of note, in pregnancy complicated by T1D, the recommendation is consuming low carbohydrate meals.

The vector of scenario parameters can be represented in the following compact form:


(20) $$\theta =(\theta_{M},\theta_{ic},\theta_{treatment},\theta_{meal})$$

#### 2.5.2 Controller Parameters

The vector of *ψ* consists of all the parameters used in zone-MPC. The cost function of zone-MPC can be shaped to obtain appropriate controller responses for various glucose behaviors. For instance, the term $G_{+}^{v}$ in the cost function can be designed to make the controller more aggressive in the case of postprandial insulin resistance that may happen during the second and third trimesters of pregnancy. The parameters in *ψ*_feedback_ include $N_{p},N_{u},G_{ZL},G_{ZH},D,G_{-}^{v},G_{+}^{v},\lambda ,\mu ,R_{+},R_{-}$ . The parameters in *ψ*_meal and correction_ include *G^m^*, *τ_c_*, *α*, that affect the meal treatment for the patient. There are other parameters for which details are beyond the scope of this paper and the interested reader is referred to the references [e.g., (17)]. In a compact form, we have:


(21) $$\psi =(\psi_{feedback},\psi_{meal\;and\;correction}).$$

### 2.6 *In-Silico* Verification Procedure for Pregnancy-Specific Zone-MPC

In order to verify the proposed tailoring of the zone-MPC, 10 *in-silico* subjects are treated with the pregnancy-specific zone-MPC under 13 different experiment conditions. The simulation duration for all experiments are 25 hours, and the closed-loop control starts one hour into the simulations. Each subject consumes three meals per day to represent breakfast, lunch, and dinner at 8 AM, 1 PM and 7 PM, respectively. Meals are equally sized as 40 grams since a relatively low carbohydrate diet is recommended during T1D pregnancy (31). Meal boluses are administered either at mealtime or 30 minutes before meals, as bolusing in advance is recommended for better postprandial glucose control in pregnancy (32). In clinical implementation, most patients with type 1 diabetes have insulin treatment parameters (i.e., *β*, CR, CF) prescribed by their health-care provider and these parameters are time dependent to address changes in insulin sensitivity. Similarly, in our simulations, these subject-specific parameters, *θ*_treatment_, are assumed clinically pre-determined and are given as inputs to the controller. The total daily insulin is also an input externally provided to the system. However, unlike the clinical implementation, *θ*_treatment_ is not time dependent in our simulations since our simulator does not capture diurnal sensitivity changes. This aspect is identical in the testing for all versions of the control algorithm. No rescue carbohydrates for hypoglycemia are administered during simulations. The simulations for each scenario are repeated ten times per subject to produce variability through random CGM noise (33) and hence, the CGM readings received by the controller are noisy. Details of the simulation scenarios are summarized in **Table 1** and explained below.

**Table 1:** Changes in parameters for each verification scenario.

Scenario	Insulin Sensitivity		Initial Glucose	Insulin Treatment Parameters			Meal Behavior		
	*V_mx_*	*kp*3	*G*_0_ (mg/dL)	*β*	*CR*	*CF*	*t*_meal_	Amt.	Adv. Bolus
A.1	–	–	90	*b*(90)	–	–	–	–	–
A.2	–	–	90	*b*(90)	–	–	–	–	✓
B.1.a	–	–	–	–	+10%	+10%	–	–	–
B.1.b	–	–	–	–	+10%	+10%	–	–	✓
B.2.a	–25%	–25%	–	–	+10%	+10%	–	–	–
B.2.b	–25%	–25%	–	–	+10%	+10%	–	–	✓
B.3.a	–	–	60	–	+10%	+10%	–	–	–
B.3.b	–	–	170	–	+10%	+10%	1h delay*	–	–
C.1	–67%	–67%	–	+50%	–50%	–33%	–	–	–
C.2	–67%	–67%	–	+50%	–	–33%	–	–	✓
D.1	+25%	+25%	60	*b*(90)	–10%	–10%	–	–	–
D.2	–25%	–25%	170	*b*(170)	+10%	+10%	–	–	–
D.3	–67%	–67%	–	+50%	–	–33%	–	–	–

“–” indicates no change from the default value:

For θ_M_, θ_ic_ and θ_treatment_ the defaults values are subject-dependent, in the simulator and percentage changes are with respect to the default values. For θ_meal_, default values are described under Section 2.6.

The function b(x) is the basal optimized to keep the glucose profile to as close to x mg/dL during fasting. In this optimization, default metabolic parameters of the in-silico subjects are used.

*The 1 hour delay is only applied to the first meal in the day.

amt. is the abbreviation for meal amount.

- **Scenario A - Treatment Regimen Adjusted for Pregnancy (Early to Mid Pregnancy)**
  This scenario provides a comparatively easy initial setup for glucose control with treatment parameters that are already well adjusted to meet the subject’s insulin requirements for pregnancy targets. To represent this condition, basal profiles of the subjects are adjusted to yield a fasting glucose value close to 90 mg/dL in the open-loop after a warm-up period. The CF and CR values are kept at their original values in the simulator that are already tuned to the subject’s metabolism (24). In this setup, we run simulations under the following conditions:

1. Meal boluses are administered at mealtimes,
2. Meal boluses are administered 30 minutes before mealtimes.

- **Scenario B - Treatment Regimen Not Adjusted for Pregnancy (Early to Mid Pregnancy)**
  Insulin requirements of women with T1D change throughout pregnancy. While the target glucose levels are significantly tighter (30), optimal adaptation of treatment parameters by expert judgment (i.e., without a formal method) may not always be possible due to metabolic changes during pregnancy. We test the pregnancy-specific zone-MPC controller’s performance when the treatment parameters, that are input to the controller, are not properly adjusted for the pregnancy specific changes in the insulin-glucose metabolism. For this purpose, (i) we use native basal profile of subjects provided in the simulator that lead to approximately 120 mg/dL fasting glucose levels, and (ii) we set both CR and CF to 110% of their nominal values for each subject. In this setup, we run simulations under the following conditions:

1. Different timing of meal boluses,
  - Meal boluses are administered at mealtimes,
  - Meal boluses are administered 30 minutes before mealtimes.
2. Additional insulin resistance is introduced *via* reducing *k_p_*_3_ and *V_mx_* by 25% of their nominal values. Under this additional resistance, we rerun our experiments for different timing of meal boluses,
  - Meal boluses are administered at mealtimes,
  - Meal boluses are administered 30 minutes before mealtimes.
3. Initial glucose levels outside the target zone,
  - Initial glucose level is around 60 mg/dL with meal bolus administered at mealtimes,
  - Initial glucose level is around 170 mg/dL. In this case, the first meal is delayed per clinical recommendations. Meal boluses are administered at mealtimes.

- **Scenario C - High Insulin Resistance with Treatment Regimen Adjusted for Pregnancy (Late Pregnancy)**

In this scenario, we test the controller performance in a setting similar to the end of pregnancy by introducing a 67% decrease in the parameters *k_p_*_3_ and *V_mx_* to induce insulin resistance (34). The treatment parameters are adjusted in accordance with the existing literature that show that the basal profile increases by 50% (35, 36), while the prescribed CR is decreased by 50% (37) and CF is decreased by 33% (38) relative to the treatment parameters in early pregnancy. In this setup, we run simulations under the following conditions:

1. Meal boluses are administered at mealtimes,
2. Meal boluses are administered 30 minutes before mealtimes.

- **Scenario D - Extreme Conditions**

The following scenarios are designed to test the controller performance when multiple challenges (e.g., initial glucose levels, subjects’ metabolic and treatment parameters) are simultaneously set to exacerbate the initial hypo-/hyper-glycemia conditions.

1. Hypoglycemia-prone setting: Initial glucose level is 60 mg/dL with basal profiles that yield a fasting glucose level of approximately 90 mg/dL. Values of *k_p_*_3_ and *V_mx_* were increased by 25% to induce higher insulin sensitivity. Both CR and CF are 10% lower; hence subjects become more prone to post-prandial hypoglycemia. Meal boluses are administered at mealtimes.
2. Hyperglycemia-prone setting: Basal profiles yield a fasting glucose level of approximately 170 mg/dL, and the initial glucose level is 170 mg/dL. Values of *k_p_*_3_ and *V_mx_* were decreased by 25% to induce insulin resistance. Both CR and CF are 10% higher, hence making subjects more prone to post-prandial hyperglycemia. Meal boluses are administered at mealtimes.
3. Late pregnancy setting with poorly adjusted CR: Pregnancy introduced changes in metabolism increase the risk of postprandial hyperglycemia in late pregnancy (39). In order to test the controller in a setting where postprandial control is more challenging compared to Scenario C.1, we introduce a 67% increase in the insulin resistance, increase the basal profile by 50%, decrease the CF by 50% in accordance with the basal profile, but keep the CR at its nominal value.

### 2.7 Tuning for Robust Performance

In this section, we formalize a robust pregnancy-specific glucose controller tuning problem as schematically summarized in **Figure 1**. In order to obtain the final design, we evaluate whether the glucose profiles obtained from the simulations adequately meet the clinical glucose control requirements.

#### 2.7.1 Problem Statement

Given a glucose profile, one can inspect whether it satisfies the clinical requirements based on metrics detailed in *Section 2.4.* to solve the following problem: *Given clinical requirements, a set of scenario parameters* Θ*, find controller parameters ψ such that closed loop glucose trajectories G satisfy the success criteria selected based on clinical requirements for all θ ∈* Θ. Note that the problem of tuning for robust performance lies in the fact that we ask a single controller parameter vector *ψ* to perform satisfactorily for all members of Θ.

#### 2.7.2 Parameter Search

Now that we have cast the pregnancy-specific glucose controller design as a parameter tuning problem, we need a tuning method. The problem is too complex to compute *ψ* in closed-form. Therefore, we rely on clinical knowledge to manually tune *ψ* and use exhaustive simulations for verification. The simulations are conducted across a wide range of scenarios and we do not expect to have one parameter vector that maximizes the primary objective across all the scenarios. Instead, our goal is to find a single *ψ* such that the success criteria, elaborated in *Section 2.4*, is met for all scenarios that the CLC system may operate in.

The tuning of *ψ* is achieved through a trial and error procedure that continues until the CLC performance is satisfactory across all scenarios, meaning that it meets the primary endpoints in Scenarios A, B, C and performs reasonably well in Scenario D. The resulting *ψ* forms the controller parameters of pregnancy-specific zone-MPC. Note that the same *ψ* is used for all subjects and all scenarios. Exemplary sensitivity analyses presented in *Section 3.5* provide further insight into the effect of tuning on performance and the ranges of parameters searched in the manual tuning process.

We note that, in our verification, we also include some *extreme scenarios* denoted by Θ_extreme_ ⊂ Θ that would be of low probability and serve as a stress-test to the controller rather than a performance test. For Θ_extreme_, captured in Scenario D, some reduction of performance is both expected and acceptable, hence we relax the clinical requirements and accept glucose profiles that are “good enough” for Θ_extreme_. Similarly, we evaluate the performance for secondary endpoints, fasting and postprandial glucose control, and aim to achieve a safe and satisfactory performance for these metrics as well.

### 2.8 Statistical Analysis

We conduct statistical analyses for each metric and scenario to evaluate the significance of the difference between controller designs, as described in the following section. The significance is evaluated based on the average outcomes per subject *via* paired t-test using two-sided p-value ≤ 0.05 significance threshold for *N* = 10. We also compare the overall performance of controllers across scenarios *via* paired t-test where each pair consists of controller outcomes under one scenario leading to an analysis with *N* = 13.

## 3 Results

First, we provide the final values obtained for the pregnancy-specific zone-MPC parameters, *ψ_p_*. Next, we compare the performance of our pregnancy-specific controller against the existing baseline. We also add to our comparisons an intermediate “zone-adjusted” controller that has only the zone-MPC ranges tuned for pregnancy for which the details are presented in the following sections. While the performance of the baseline controller is of interest as it is the existing and clinically-validated design, a comparison of the zone-adjusted versus our pregnancy-specific controller is more relevant since these two controllers have the same target zones. The results are evaluated mainly for the verification of the pregnancy-specific design but they also provide an insight on whether a departure from the baseline design was necessary for more favorable glucose control to achieve pregnancy-specific targets. Finally, we specifically focus on pregnancy-specific controller performance and explore its qualitative robustness through scenarios featuring a variety of challenges.

### 3.1 Pregnancy-Specific Glucose Controller Parameters

In order to accommodate the major changes that occur in the glucose metabolism and glucose control requirements in pregnancy with T1D, the following design choices were made and are summarized in **Table 2**. For each parameter, the starting values are the values used in the baseline controller that are provided in **Table 2**.

**Table 2:** Parameters in Baseline vs. Pregnancy-Specific Zone-MPC.

Design Element	Symbol	Baseline Value	Pregnancy Value	Effect of the Change
**Zone-MPC**				
Day-time target glucose zone	*G_ZL_* - *G_ZH_*	90-120 mg/dL	80-110 mg/dL	Tighter glucose control as recommended for pregnant women with diabetes
Night-time target glucose zone	*G_ZL_* - *G_ZH_*	100-120 mg/dL	80-100 mg/dL	Tighter glucose control as recommended for pregnant women with diabetes
Reference fasting glucose	*G*_ref_	110 mg/dL	90 mg/dL	Controller deviation variables are calculated using a lower glucose value in the center of the zone
Active glucose velocity-penalty range	${\text{G}}_{\_}^{v}$ - ${\text{G}}_{+}^{v}$	140-180 mg/dL	120-180 mg/dL	Reduced post-prandial glucose exposure as recommended for pregnant women with diabetes
Meal bolus insulin decay curve	*λ*	4 hours	3 hours	Earlier relaxation of the insulin on board related constraint on controller action
**Meal and Correction Control**				
Additional correction bolus threshold (added to the meal bolus)	*τ_c_*	150 mg/dL	100 mg/dL	Reduced post-prandial glucose exposure with more assertive meal control strategy as recommended for pregnant women with diabetes
Target glucose in corrections	${\text{G}}_{\text{ref}}^{\text{corr}}$	150 mg/dL	90 mg/dL	More assertive hyperglycemia response integrated through correction bolus
Glucose threshold for reducing meal bolus	*G^m^*	120 mg/dL	70 mg/dL	Reduced post-prandial glucose exposure as recommended for pregnant women with diabetes

- First, we modify the reference glucose and target zones.

1. [*G_ZL_*, *G_ZH_*] defines the target control zone. Thus, they are chosen to be customized for tighter targets in pregnancy. These zones are tuned separately for the day and night times. We adjusted both for tighter glucose control requirements during pregnancy.

2. *G_ref_* is the glucose reference and is used both in predicting glucose trajectory and calculating the optimal insulin input. *G_ref_* is also the reference value mentioned in the computation of total insulin required to bring the glucose level to reference in (17). We decreased the reference fasting glucose level to 90 mg/dL, aligned with the updated target zones and target fasting glucose level for pregnancy.

- In accordance with the tighter glucose control requirements overall and the shift in the target glucose zones, we made the following adjustments to the meal and correction bolus calculation to proactively counter the increased risk of postprandial hyperglycemia with advancing gestation (39):

3. *τ_c_* defines the threshold for adding correction to meal boluses. By setting *τ_c_* lower, the meal control strategy can be made more protective against postprandial hyperglycemia. We decreased the threshold for adding a correction component to the meal bolus from 150 mg/dL to 100 mg/dL.

4. $G_{\text{ref}}^{\text{corr}}$ is the target glucose level used in the correction calculation. In order to achieve lower fasting glucose and more intense postprandial glucose control, $G_{\text{ref}}^{\text{corr}}$ is tuned to a lower value (i.e., 90 mg/dL) than the baseline controller. We select the $G_{\text{ref}}^{\text{corr}}$ in the middle of the tighter nighttime target zone.

5. *G^m^* defines the glucose threshold for full meal bolus and below which the bolus is reduced *via* multiplying by a factor, *α*, which is set to 0.8 in baseline zone-MPC. By setting *G^m^* to lower values, more proactive action against postprandial hyperglycemia is obtained. The threshold for reducing the meal bolus is decreased from 120 mg/dL to 70 mg/dL.

- Finally, we tuned the following parameters to give more leeway to the controller and keep one to two hour postprandial glucose within the target range as recommended:

6. *λ* defines the choice of IOB decay curve, hence affects the resulting IOB in (16). The shorter the decay curve is, the earlier the controller is relaxed to inject additional insulin. By this adjustment, a more assertive response to postprandial hyperglycemia can be obtained. We relaxed the meal-related IOB constraint by using a decay curve of three hours as opposed to the baseline value of four hours.

7. $\left[G_{\_}^{v},G_{+}^{v}\right]$ defines the glucose interval where additional penalty is added to increasing glucose for more aggressive response against hyperglycemia. By tuning this interval, we can adjust the assertiveness of the CLC response to hyperglycemia. We decreased the glucose lower-bound for increased velocity-penalty from 140 mg/dL to 120 mg/dL. We kept the glucose upper-bound for increased velocity penalty at 180 mg/dL.

Note that other parameters in *ψ* such as *R*_+_, *R*_–_, *N_p_*, *N_u_* could also be modified but we conjecture that varying the parameters above is enough to shape the target controller behavior in providing acceptable performance and keeps the tuning problem tractable.

### 3.2 Baseline, Zone-Adjusted, and Pregnancy-Specific Designs

As pregnancy is associated with lower target glucose ranges, we test if lowering the zones in baseline zone-MPC is sufficient to obtain a satisfactory pregnancy-specific controller, and whether changes to other parameters in *Section 3.1* are necessary. In order to have a relevant comparison, we introduce a zone-adjusted controller where only the target zones are adjusted according to pregnancy requirements. We call this controller “zone-adjusted”. In summary, we have the following controllers:

- Baseline: The standard zone-MPC that is tuned for and has been successfully tested on non-pregnant individuals. The values in *ψ_Baseline_* are available in (17) and (21).
- Zone-Adjusted: This is a version of the zone-MPC that shares the same features and parameter values with the baseline, except that the values of *G_ZL_*, *G_ZH_*, *G*_ref_ are shifted down to adapt to the lower pregnancy glucose targets with the corresponding values provided in *Section 3.1*.
- Pregnancy-Specific: As mentioned earlier, *G_ZL_*, *G_ZH_*, *G*_ref_, *τ_c_*, *G^m^*, $G_{\text{ref}}^{\text{corr}}$ , λ , $G_{\_}^{v}$ , $G_{+}^{v}$ are tuned for pregnancy with the values provided in *Section 3.1*.

### 3.3 Time in Range Performance

#### 3.3.1 Baseline vs. Zone-Adjusted vs. Pregnancy-Specific Zone-MPC Performance

We compare the performance of each controller for each scenario and each glycemic metric. The average outcomes across *in-silico* subjects are presented for pregnancy-specific glycemic ranges and for non-pregnant adult glycemic ranges in **Table 3** and **Table 4**, respectively. The colors depict the performance with continuous shades from green (highest performance) to red (poorest performance). Note that the relation of colors to numbers is metric-dependent. For instance, for time percentage in 63 - 140 mg/dL (the target for pregnancy), higher values are desirable whereas for time percentage > 180 mg/dL (hyperglycemia threshold for non-pregnant), lower values are sought.

**Table 3:** Glucose control performances of different designs evaluated for pregnancy glycemic targets across all *in-silico* subjects.

Scenario	% Time *<* 54 mg/dL			% Time *<* 63 mg/dL			% Time 63-140 mg/dL			% Time *>* 140 mg/dL		
	BL	ZA	PS	BL	ZA	PS	BL	ZA	PS	BL	ZA	PS
A.1	0.03	0.05	0.16	0.06	**0.26**	**0.58**	85.32	90.67	91.34	14.63	**9.07**	**8.08**
A.2	0.04	0.07	0.11	0.07	0.42	0.61	89.9	94.33	95.20	10.03	5.25	4.19
B.1.a	0.01	0.03	0.05	0.03	0.05	0.22	76.19	**83.14**	**86.78**	23.79	**16.81**	**13.00**
B.1.b	0.03	0.03	0.04	0.03	0.10	0.16	79.24	**86.9**	**91.72**	20.72	**13.00**	**8.12**
B.2.a	0.00	0.00	0.00	0.00	0.00	0.02	61.78	**69.77**	**76.36**	38.22	**30.23**	**23.63**
B.2.b	0.00	0.00	0.01	0.00	0.03	0.03	63.48	**71.89**	**79.88**	36.52	**28.08**	**20.10**
B.3.a	0.45	0.48	0.51	1.77	**1.82**	**1.99**	75.65	**82.72**	**84.84**	22.58	**15.46**	**13.17**
B.3.b	0.00	0.02	0.04	0.01	0.05	0.22	69.82	**77.53**	**81.36**	30.17	**22.42**	**18.42**
C.1	0.00	0.02	0.02	0.00	0.06	0.05	67.96	**70.51**	**76.3**	32.04	**29.43**	**23.66**
C.2	0.00	0.02	0.02	0.00	0.06	0.07	69.65	**72.47**	**79.9**	30.35	**27.47**	**20.02**
D.1	2.09	2.43	3.17	3.95	**5.17**	**6.78**	87.63	**89.86**	**88.86**	8.42	4.97	4.36
D.2	0.00	0.00	0.00	0.00	0.00	0.01	33.9	**46.15**	**55.47**	66.1	**53.85**	**44.52**
D.3	0.00	0.02	0.02	0.00	0.06	0.05	56.76	**58.78**	**63.4**	43.24	**41.16**	**36.55**

Poorest Performance 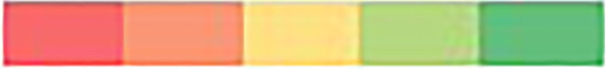 Highest Performance*

BL, Baseline; ZA, Zone-Adjusted; PS, Pregnancy Specific.

*Color codes are applied separately for each glycemic metric to help the evaluation of performances across scenarios and designs.

Statistically significant differences between zone-adjusted and pregnancy-specific controller performances are marked with bold. P-values for all metrics and scenarios can be found in the **Supplementary Material**.

**Table 4:** Glucose control performances of different designs evaluated for standard glycemic targets across all *in-silico* subjects.

Scenario	% Time *<* 70 mg/dL				% Time 70-180 mg/dL				% Time *>* 180 mg/dL				% Time *>* 250 mg/dL		
	BL	ZA	PS		BL	ZA	PS		BL	ZA	PS		BL	ZA	PS
A.1	0.17	** 1.09**	** 1.74**		99.01	98.57	98.1		0.83	0.34	0.16		0.00	0.00	0.00
A.2	0.24	** 1.49**	** 2.04**		99.42	** 98.37**	** 97.89**		0.34	0.14	0.06		0.00	0.00	0.00
B.1.a	0.04	** 0.36**	** 0.71**		98.63	98.96	98.93		1.34	0.68	0.36		0.00	0.00	0.00
B.1.b	0.04	** 0.41**	** 0.63**		99.29	99.29	99.25		0.67	0.3	0.12		0.00	0.00	0.00
B.2.a	0.00	0.06	0.08		96.19	97.57	98.83		3.81	** 2.37**	** 1.09**		0.01	0.00	0.00
B.2.b	0.02	0.07	0.08		97.33	98.44	99.25		2.65	** 1.5**	** 0.67**		0.00	0.00	0.00
B.3.a	3.04	** 3.39**	** 3.66**		95.38	95.81	95.80		1.58	0.79	0.54		0.00	0.00	0.00
B.3.b	0.03	** 0.30**	** 0.74**		98.58	98.69	98.45		1.40	1.00	0.81		0.00	0.00	0.00
C.1	0.02	0.18	0.18		98.04	** 98.34**	** 99.13**		1.94	** 1.48**	** 0.69**		0.00	0.00	0.00
C.2	0.03	0.24	0.30		98.76	** 98.79**	** 99.3**		1.21	** 0.97**	** 0.40**		0.00	0.00	0.00
D.1	5.56	** 8.00**	** 10.46**		94.14	** 91.86**	** 89.49**		0.30	0.14	0.05		0.00	0.00	0.00
D.2	0.00	0.00	0.03		85.48	** 89.99**	** 94.77**		14.52	** 10.01**	** 5.20**		0.02	0.01	0.01
D.3	0.02	0.20	0.18		91.64	** 92.90**	** 95.59**		8.34	** 6.89**	** 4.23**		0.01	0.00	0.00

Poorest Performance 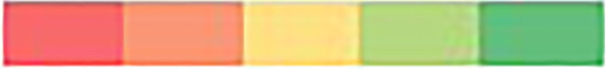 Highest Performance*

BL, Baseline; ZA, Zone-Adjusted; PS, Pregnancy Specific.

*Color codes are applied separately for each glycemic metric to help the evaluation of performances across scenarios and designs.

Statistically significant differences between zone-adjusted and pregnancy-specific controller performances are marked with bold. P-values for all metrics and scenarios can be found in the **Supplementary Material**.

The results for pregnancy specific ranges are in **Table 3**. The pregnancy-specific design satisfies the pregnancy-specific consensus glycemic targets in all scenarios and for all metrics. Likewise, the baseline zone-MPC satisfies the standard consensus glycemic targets for the same scenarios and for all metrics (**Table 4**). Summary glucose-insulin plots for each scenario and controller are provided in the **Supplementary Material**. As one can inspect, the performance for the time in and above the 63 - 140 mg/dL range steadily increase from the baseline controller to the zone-adjusted version and then to the pregnancy-specific zone-MPC. Across 13 scenarios, pregnancy-specific zone-MPC leads to a 10.3 ± 5.3% increase in the time in pregnancy target range (baseline zone-MPC: 70.6 ± 15.0%, pregnancy-specific zone-MPC: 80.8 ± 11.3%, *p* < 0.001) and a 10.7 ± 4.8% reduction in the time above the target range (baseline zone-MPC: 29.0 ± 15.4%, pregnancy-specific zone-MPC: 18.3 ± 12.0, *p* < 0.001). There is no significant difference in the time below range between the controllers (baseline zone-MPC: 0.5 ± 1.2%, pregnancy-specific zone-MPC: 3.5 ± 1.9%, *p* = 0.1). The outcomes that are significantly different between zone-adjusted and pregnancy-specific controllers are highlighted with bold in **Table 3** and **Table 4**.

The pregnancy-specific design outperforms the zone-adjusted one significantly in time in and above the 63 - 140 mg/dL range in 10 out 13 scenarios. The exact p-values are provided in the **Supplementary Material**. For the time below target range (< 63 mg/dL), we observe that the zone-adjusted controller significantly outperforms the pregnancy-specific controller in three scenarios, including an extreme one. Note that the absolute difference between the corresponding numbers are clinically less significant as the increases in time below 63 mg/dL are less than 5 minutes in 24h (0.32%) except for extreme scenario D.1 where the increase is about 23 minutes (1.61%). Equally important, all designs proved high protection against very low - below 54 mg/dL - and very high - above 250 mg/dL - glucose. Next, we explore the effect of each scenario only on the pregnancy-specific zone-MPC.

#### 3.3.2 Pregnancy-Specific Zone-MPC Performance

**Figure 2:**
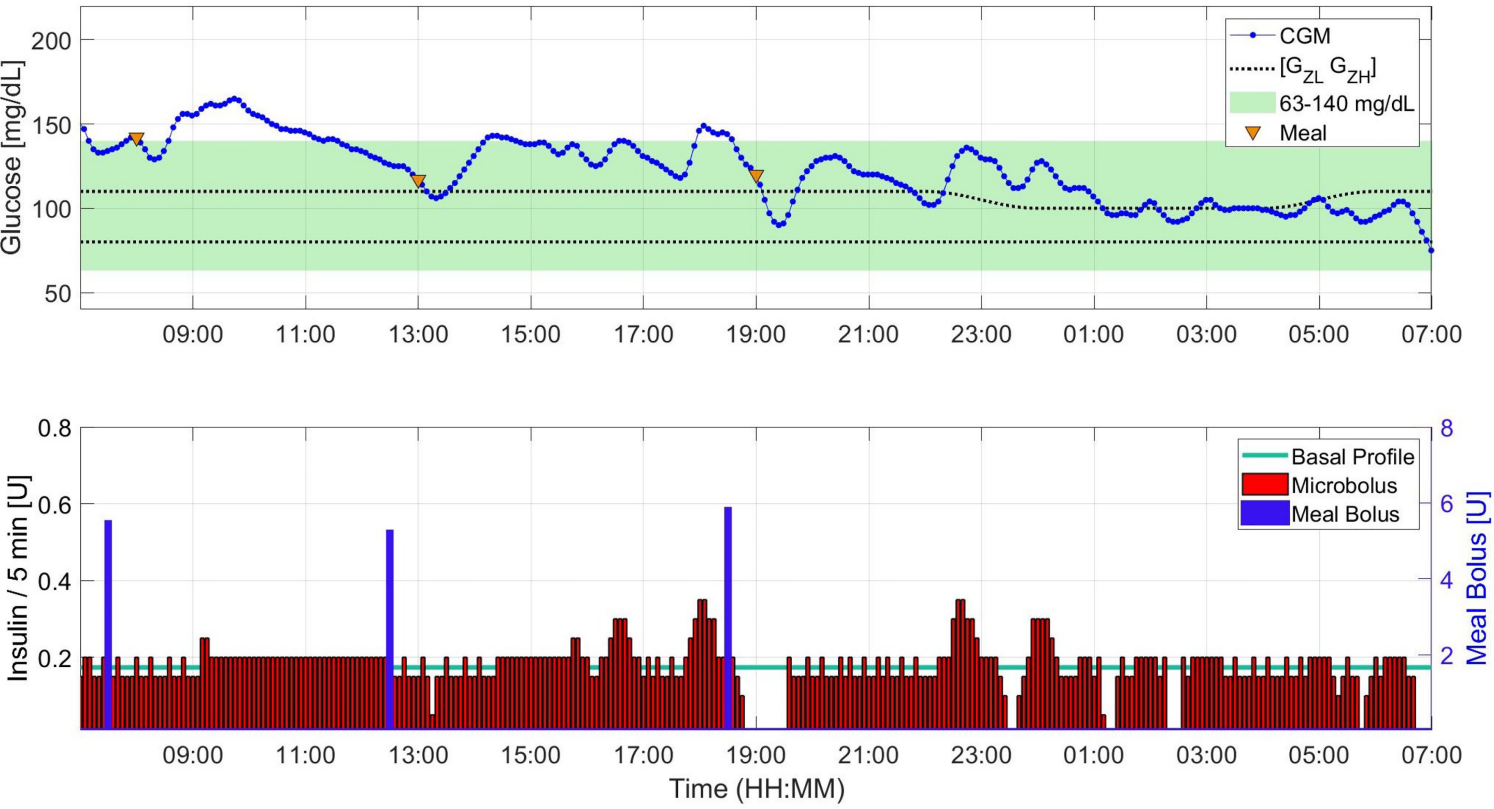
Sample insulin input, meal intake, and glucose trajectory for one subject under Scenario C.2.

- **Scenario A - Treatment Regimen Adjusted for Pregnancy:** The average percentage of time spent within the target range is over 90% in both bolusing strategies. This percentage is higher when meal boluses are administered 30 minutes before meal intake compared to meal boluses administered at mealtimes. Between the two bolusing strategies, the most pronounced difference is in the time spent in hyperglycemia over 24h. Bolusing in advance decreased the time spent above the target range from about 8% to about 4%. For both strategies, the average percentage of time spent in hypoglycemia is less than 1% similar to the time spent above 180 mg/dL. The average percentage of time above 250 mg/dL is 0%. Average total daily insulin (TDI) and daily average glucose are 40.1 U and 111.1 mg/dL, respectively.
- **Scenario B - Treatment Regimen Not Adjusted for Pregnancy (Early to Mid Pregnancy):** Due to sub-optimal treatment parameters, both time above 140 mg/dL and 180 mg/dL are higher compared to the respective observations under Scenario A. This leads to a lower percentage of time spent in the 63 - 140 mg/dL target range. However, the average percentage of time spent within this range remains over 75% with a higher performance when meal boluses are administered 30 minutes before meal intake compared to meal boluses administered at mealtimes. Between the two bolusing strategies, a pronounced difference is observed in the time spent in hyperglycemia. The strategy of bolusing in advance reduces some of the time spent above the target range. Between Scenario B.1.a and B.3.b the only difference is the initial glucose value (~120 vs. 170 mg/dL) and the results show more than 5% difference in the 63-140 mg/dL range, despite the first meal being delayed one hour not to exacerbate the high glucose risk according to the clinical guidelines. This observation suggests that “too high” starting glucose value is a challenge to the daily (i.e., 24h) glucose control performance. In all settings of Scenario B, the average percentage of time spent below 54 mg/dL is less than 1%, time below 63 mg/dL is less than 4%, and time above 180 mg/dL never exceeds 1.1%. The average percentage of time above 250 mg/dL is approximately 0% throughout. For Scenario B.1, average TDI was 39.2 U and daily average glucose was 119.8 mg/dL. In Scenario B.2, we obtain an average glucose of 129.2 mg/dL with 41.9 U TDI. In Scenario B.3.a, low initial glucose values lead to a lower TDI, 38.1 U, and the average glucose is 117.5 mg/dL. Finally for Scenario B.3.b, TDI is 40 U and average glucose is 123.7 mg/dL.
- **Scenario C - High Insulin Resistance (Late Pregnancy Conditions):** Due to the significantly low insulin sensitivity, subjects have a higher risk of hyperglycemia. Despite this increased risk, the percentages of time spent in and above the 63 – 140 mg/dL target range are similar to the outcomes in scenario B.2. This observation shows that with insulin treatment parameters adjusted according to the literature, without the need for personalized fine tuning, pregnancy-specific zone-MPC can perform well in insulin resistance conditions during late pregnancy. The impact of bolusing in advance is also similar to the impact seen in Scenario B.2, as it shifts the time in the target range 3% above through reduction of time above the target range. We provide a sample insulin-glucose trajectory for Scenario C.2 in **Figure 2**. From Scenario A.2 to Scenario C.2, TDI increases on average 74 ± 12% (minimum: 51%, and maximum: 93% increase) aligned with the data from the literature (8, 35). For Scenario C, TDI is 68.8 U and daily average glucose is 126.1 mg/dL.
- **Scenario D - Extreme Conditions:** As expected, the average percentage of time spent in hypoglycemia in D.1, 6.8%, is the highest in this category among all the experiments conducted. The amount of time spent below 54 mg/dL is 3.2% and this is the only experiment with a result that violates the consensus thresholds for hypoglycemia related metrics. Scenario D.2 yields the lowest average percentage of time spent within the target range, 55.5%. Unlike D.1, there is no time spent in hypoglycemia under D.2. However, the average percentage of time spent in hyperglycemia is 44.5%, and marks as the poorest outcome in this category among all the experiments conducted with pregnancy-specific zone-MPC. This is the only scenario that the outcome exceeds the consensus threshold for time above 140 mg/dL. In Scenario D.3, when the poorly adjusted CR causes a decrease in the time in range, pregnancy-specific zone-MPC performs significantly higher than the zone-adjusted controller.

### 3.4 Fasting and Postprandial Control Performance

Fasting glucose is computed as the average glucose between 6 AM- 7AM following the night with CLC and no food intake after 7 PM. The aim is to have fasting values between 63 – 95 mg/dL. The deviations from the upper and lower parts of this range are not treated equally; deviations above 95 mg/dL are permitted in an exchange for performance while deviations below 63 mg/dL pose a higher risk due to the asymmetrical clinicial risks associated with hypo/hyper-glycemia (16). Early postprandial glucose control performance is evaluated through time spent in the target range of 63 - 140 mg/dL within the two hours of the meal intake.

#### 3.4.1 Design Comparisons

**Figure 3:**
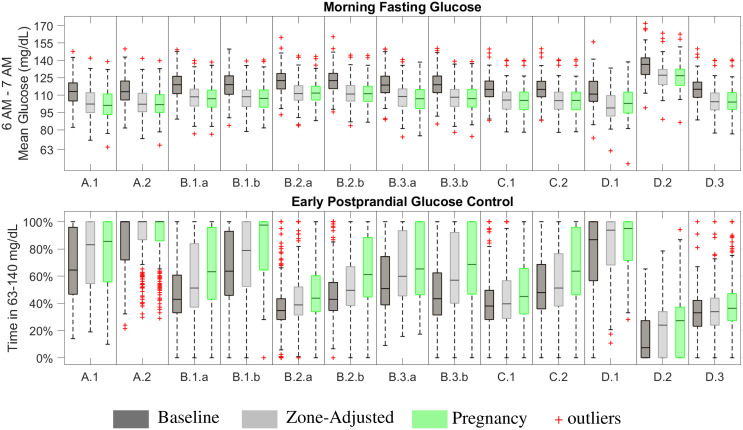
Fasting and two-hour postprandial glucose control performance.

The main variables affecting fasting glucose outcomes are insulin sensitivity and *u*_basal_. As long as the *u*_basal_ is able to keep the glucose within the target zone, [*G_ZL_*, *G_ZH_*], controller commands *u*_basal_. When the *u*_basal_ is not able to keep the glucose within the target zone, the controller adjusts the injected insulin amount in a way that the resulting glucose stays in or as close as it can get to the target zone. As a result, fasting glucose performances are similar across scenarios for zone-adjusted and pregnancy-specific controllers while it is higher in the results with the baseline zone-MPC (**Figure 3**). On the other hand, results for early postprandial glucose control performance show an increasing trend from baseline to zone-adjusted and then to pregnancy-specific controller. These results support our hypothesis that tuning controller parameters beyond the target zones is worthwhile for customization to pregnancy-specific targets.

#### 3.4.2 Pregnancy-Specific Zone-MPC: Fasting Performance

The pregnancy-specific design outperforms the baseline design in achieving levels closer to 95 mg/dL but shows similar performance to the zone-adjusted controller, assessed by the median outcomes (median [Q1,Q3]) for each scenario (**Figure 3**), while not meeting this secondary endpoint in many cases. The highest performance is obtained with the pregnancy-specific design under Scenarios A.1 and A.2. Resulting CGM measured fasting glucose levels are 101.2 [93.3,110.9] mg/dL and 101.7 [95.0,110.3] mg/dL, respectively. While *u*_basal_ is set to yield approximately 90 mg/dL average fasting intravenous glucose in the open-loop for all subjects in these scenarios. CGM noise, injecting micro-boluses every five-minutes instead of every minute as implemented in the *in-silico* open-loop setting, and wider zones starting from 6 AM on (i.e., 80 - 110 mg/dL) contribute to the variability and higher than 95 mg/dL glucose levels observed in the fasting outcomes. The poorest performance occurs in D.2, where each subject’s *u*_basal_ is set to yield 170 mg/dL fasting glucose in the open-loop control. CLC manages to reduce the effect of sub-optimal basal profiles to some extent and the resulting fasting glucose is 126.7 [118.4, 132.4] mg/dL. The reasons behind most of the scenarios yielding similar fasting outcomes are as follows: (i) the meal and initial condition related challenges do not affect the overnight fasting outcomes, (ii) the controller is able to overcome challenges related to insulin sensitivity and sub-optimal treatment parameters overnight as long as they are not extreme or can be addressed by decreasing injected insulin amount, as in Scenario D.1. Due to safety considerations, cases like Scenario D.2 can be mitigated by insulin injections higher than *u*_basal_ only up to a certain level.

#### 3.4.3 Pregnancy-Specific Zone-MPC: Postprandial Glucose Control Performance

Since postprandial glucose control is affected by starting glucose at the meal time and the outcomes include all three meals consumed in the day, results are highly variable. Especially for the first meal of the simulation, consumed one-hour into the CLC, the controller can mitigate the sub-optimal initial glucose only to some extent. The highest performance, median TIR of 100%, is achieved in Scenario A.2 where the insulin treatment parameters are adjusted for pregnancy targets and meal boluses are injected in advance. Advanced meal-bolusing improves postprandial control to the extent that the runner-up performance is achieved under Scenario B.1.b despite sub-optimal treatment parameters. Increased postprandial time in 63 - 140 mg/dL seen in C.2 compared to C.1 also is another example where the importance of advance bolusing for better meal control is emphasized. Meal intake is well-controlled in Scenario D.1 as carbohydrate ingestion helps mitigating the extreme hypoglycemic conditions in this scenario. Results for B.1.a, B.2.b, B.3.a, B.3.b, and C.2 are comparable. The poorest meal control occurs in D.2. as expected since meal intake further exacerbates challenges due to the extreme hyperglycemic conditions in this scenario.

### 3.5 *Post-Hoc* Evaluation of Parameter Choices

Here, we show how other choices of *ψ* affect the glucose outcomes. The number of dimensions in *ψ*, clinical metrics, and scenarios make a thorough visualization impossible. Therefore, we provide partial visualizations by holding all but two specific elements in *ψ* as in *ψ_p_* and illustrate the changes in glucose time in range metrics.

For this purpose, we provide an exemplary *post-hoc* analysis on the effect of using different [*G*_ZL_, *G*_ZH_] under two scenarios that are of high likelihood to occur in real life: Scenario A.2 and Scenario B.1.a. Similar analyses for other elements in *ψ* are provided in **Supplementary Material** for the same scenarios. We choose the range as 70 - 90 mg/dL for *G*_ZL_ and 80 - 130 mg/dL for *G*_ZH_. We restrict *G*_ZH_ to be not lower than *G*_ZL_ + 10 mg/dL to obtain proper zones. Within the search interval, a grid search is performed for all combinations with an increment of 10 mg/dL for each element of the pair [*G*_ZL_, *G*_ZH_]. All other parameters are set at their values in *ψ_p_*. As this analysis requires exhaustive simulations, we ran simulations with 10 subjects without repetition and kept the nighttime target zones the same as the daytime target zones for simplicity. Heat-maps are used to illustrate the changes in the glucose time in, below, and above the target range for the search interval (**Table 5** and **Table 6**).

**Table 5:** *Post-hoc* parameter choice evaluation under Scenario A.2.

**63-140 mg/dL**							
*G*_**ZH**_ * G*_**ZL**_		**80**	**90**	**100☾**	**110 ☼**	**120**	**130**
** 70**		95.27%	94.38%	95.69%	94.93%	95.32%	95.52%
** 80 ☼☾**		NA	95.86%	96.20%	95.98%	95.40%	95.39%
** 90**		NA	NA	95.55%	95.18%	94.25%	93.32%
**< 63 mg/dL**							
*G*_**ZH**_ * G*_**ZL**_		**80**	**90**	**100☾**	**110 ☼**	**120**	**130**
** 70**		2.66%	2.77%	1.47%	1.54%	1.28%	1.15%
** 80 ☼☾**		NA	0.97%	0.46%	0.19%	0.10%	0.01%
** 90**		NA	NA	0.03%	0.00%	0.01%	0.01%
**> 140 mg/dL**							
*G*_**ZH**_ * G*_**ZL**_		**80**	**90**	**100☾**	**110 ☼**	**120**	**130**
** 70**		2.07%	2.85%	2.84%	3.53%	3.39%	3.33%
** 80 ☼☾**		NA	3.16%	3.33%	3.83%	4.50%	4.60%
** 90**		NA	NA	4.42%	4.82%	5.73%	6.68%

◼ values inΨp ☼: Daytime ☾: NIght time

Poorest Performance 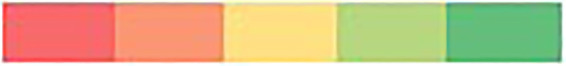 Highest Performance*

*Color codes are applied separately for each glycemic metric and are meant to help the evaluation of performances across parameter selections within each scenario.

**Table 6:** *Post-hoc* parameter choice evaluation under Scenario B.1.a.

**63-140 mg/dL**						
*G*_**ZH**_ * G*_**ZL**_	**80**	**90**	**100☾**	**110 ☼**	**120**	**130**
** 70**	90.45%	90.49%	89.65%	89.14%	87.91%	87.95%
** 80 ☼☾**	NA	89.54%	87.99%	87.08%	86.73%	85.95%
** 90**	NA	NA	86.63%	84.77%	83.23%	81.80%
**< 63 mg/dL**						
*G*_**ZH**_ * G*_**ZL**_	**80**	**90**	**100☾**	**110 ☼**	**120**	**130**
** 70**	0.76%	0.35%	0.34%	0.32%	0.27%	0.13%
** 80 ☼☾**	NA	0.00%	0.00%	0.00%	0.00%	0.00%
** 90**	NA	NA	0.00%	0.00%	0.00%	0.00%
**> 140 mg/dL**						
*G*_**ZH**_ * G*_**ZL**_	**80**	**90**	**100☾**	**110 ☼**	**120**	**130**
** 70**	8.79%	9.17%	10.01%	10.54%	11.82%	11.92%
** 80 ☼☾**	NA	10.46%	12.01%	12.92%	13.27%	14.05%
** 90**	NA	NA	13.37%	15.23%	16.77%	18.20%

◼ values inΨp ☼: Daytime ☾: NIght time

Poorest Performance 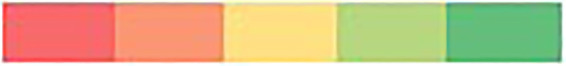 Highest Performance*

*Color codes are applied separately for each glycemic metric and are meant to help the evaluation of performances across parameter selections within each scenario.

When the treatment parameters are already adjusted for pregnancy, TIR outcomes are all above 93% and show small changes across the search interval (**Table 5**). However, the pattern of changes guide the search towards [80,100] mg/dL from both sides for increasing TIR. This zone corresponds to the nighttime zones in *ψ_p_* and the selected daytime zone, [80,110] mg/dL has a similar TIR performance. Lower values for both *G*_ZL_ and *G*_ZH_ come with a cost on the time below target and higher values come with a cost of time above the target.

As for Scenario B.1.a, where treatment parameters are not adjusted for pregnancy (i.e, making subjects more prone to hyperglycemia) changes in the glucose control performance for different target zones are more pronounced than the ones observed in Scenario.A.2. Higher values of [*G*_ZL_, *G*_ZH_] lead to less favorable TIR outcomes due to increasing time above 140 mg/dL. While the search interval for *G*_ZL_ includes 70 mg/dL for evaluation of outcomes in both directions, glucose control below 80 mg/dL is not recommended because too tight control restricts fetal development (29). As expected, time below the target range stays low to non-existent throughout under this scenario (**Table 6**).

Exemplary analyses on local sensitivity of metrics around *ψ_p_* under the same scenarios for selected pairs of elements in *ψ* are provided in the **Supplementary Material**. In these analyses, the range of evaluation for each parameter is determined in a way that surround plausible values: between 70 - 110 mg/dL for *G*_ref_, 1 to 6 hours for *λ*, between 90 and 130 mg/dL for *τ_c_*, between 110 and 140 mg/dL for $G_{\_}^{v}$ , and from 110 to 220 mg/dL for $G_{+}^{v}$ . These analyses provide insight into how the changes in glycemic outcomes for various parameter values differ across scenarios. We note that the final parameter selection presented in **Table 2** is not the optimal selection for any specific scenario. Instead, it strikes a balance between the clinical recommendations for glycemic control over the selected range of scenarios as explained in *Section 2.7*.

## 4 Discussion

### 4.1 Significance

In this work, we tailored a zone-MPC controller to meet the tight glucose control requirements in pregnant women with T1D. We verified the proposed pregnancy-specific CLC system under a broad range of clinically possible scenarios. To provide insight on the performance of the baseline controller and the impact of tailoring, we performed the same experiments with the baseline and only zone-adjusted controller designs as well. The baseline zone-MPC design, aimed to succeed in non-pregnancy glucose target range of 70 – 180 mg/dL, showed a performance above 95% time in this range for all but extreme case scenarios explored in this study. The high percentage of time in the non-pregnancy target range obtained by the baseline zone-MPC is consistent with some of the previous *in-silico* studies on zone-MPC (40, 41) Both studies reported above 90% time in 70 - 180 mg/dL while the meals in their scenarios had higher carbohydrate content (i.e., ranging from 50 to 100 grams per meal) compared to our experiments (i.e., 40 grams per meal). In our work, low carbohydrate content of the meals, all meals being announced at or before their intake and accurately, the effect of titrating the insulin treatment parameters to achieve pregnancy-specific glucose control requirements in Scenario A and partially in Scenario C contributed to the high performance of all three controllers. Here, we note that the choice of low carbohydrate intake was in accordance with the clinical guidelines for pregnancy complicated by T1D. Pregnant women with T1D have much stricter dietary plans compared to their non-pregnant counterparts due to the additional risks that high glucose poses for the mother and the fetus. With this awareness, this is a particularly motivated population to follow the clinical recommendations that include a low carbohydrate diet.

As the baseline controller provided a strong foundation for our tailoring purpose, the natural question was whether one could achieve the pregnancy specific glucose targets by simply shifting the zones down and without further tuning other parameters. To answer this question, we had the zone-adjusted MPC which was a naively tuned version of the baseline controller where the target zone and reference glucose are adapted to the pregnancy targets but other parameters stayed the same as the baseline zone-MPC. Our results showed that the overall performance of the zone-adjusted controller mostly falls between baseline and pregnancy-specific designs. It is worth to note that while the zone-adjusted design also meets the primary endpoint in most cases, the improvement is significant as clinical studies suggest that even a 5% improvement in TIR is associated with a reduction of birth complications (42). The difference between the zone-adjusted and pregnancy-specific designs are significant in multiple cases, implying that tuning parameters in *Section 3.1* beyond the target zone have a meaningful effect. While there was no significant difference in the time below 63 mg/dL across scenarios, in three out of 13 scenarios, pregnancy-specific zone-MPC led to a significant increase in the time below range. We consider this as an acceptable trade off since the improvement in other metrics were significant and the degradation in time below < 63 mg/dL was small - the difference ranged from 0.01% to 0.54% (from 1.4 to 7.8 minutes), except in the extreme case scenario D.1 that we discuss under *Section 4.3*.

Among the presented simulation scenarios, the highest glucose control performance was obtained under Scenario A with above 91% time in the target range. Although sub-optimalities introduced under Scenario B decreased the control performance, the glucose outcomes satisfied the primary endpoint of our work. The extreme case scenarios presented in Scenario D showed that the lowest performance occurred when multiple conditions were combined to increase the risk of hyperglycemia. In Scenario C (akin to the end of third trimester in pregnancy), we observe that the primary endpoint is still met by the pregnancy-specific controller. While it needs further studies and clinical validation, the results in **Table 3** suggest that our controller is robust enough to be deployed for pregnancy.

### 4.2 Clinical Trial

Our work in this manuscript used 10 *in-silico* subjects for guiding the customization of baseline zone-MPC to pregnancy. Resulting satisfactory performance enabled us to move to testing our system in a clinical trial. The pregnancy-specific controller is currently under clinical evaluation in a FDA-approved outpatient clinical study (NCT04492566). The results from our pilot clinical study on 8 subjects over 48 hours of use indicate safety and feasibility of the pregnancy-specific zone-MPC. In particular, time in the pregnancy target range was 79.3%, time < 63 mg/dL was 1.6%, and time < 140 mg/dL was 19.1% even though there were no restrictions to the meal intake or physical activity of the participants (43). Testing of the controller in the home-setting continues at this time.

### 4.3 Limitations and Future Work

A limitation of our work was due to the fact that changes in glucose-insulin-meal metabolism being yet to be precisely quantified and hence, absence of related mathematical models for T1D during pregnancy. Therefore, we used scenario parameters to simulate *pregnancy-like* metabolic, clinical and behavioral conditions, such as changing insulin sensitivities, insulin treatment parameters, meal intake and bolusing behavior. Future research that focuses on modeling the glucose-insulin metabolism during pregnancy could facilitate building simulation platforms tailored to this cohort, which would facilitate the development of optimal insulin treatment strategies and systems for this cohort. While we tried to cover a comprehensive range of potential scenarios in our simulations, we note that our simulations do not capture diurnal sensitivity changes, other changes to metabolism during pregnancy due to insulin pharmacokinetics and body weight. Furthermore, the diversity of subjects captured in the *in-silico* experiments are inevitably less than the diversity of a realistic population in different aspects (e.g., the severity of diabetes and demographic profiles), and not all challenges that may occur in real life and affect the glucose control performance of any CLC system are simulated (e.g., physical activity, large meals, missed meals/boluses, stress). Especially under conditions that are particularly prone to hypoglycemia, such as engaging in a long physical activity session, the importance of preventative measures against low glucose would be elevated when using a pregnancy-specific controller. This is due to the already low target for the glucose levels compared to a standard controller, that might increase the risk of hypoglycemia (e.g., results for Scenario D.1). This is an acceptable trade-off to achieve lower glucose levels.

Although the strength of our approach is in offering an *off-line* tuning strategy for robust performance that leads to a single controller capable of achieving satisfactory control throughout pregnancy, an accompanying limitation is that we rely on having insulin treatment parameters prescribed *a priori* (i.e., *θ*_treatment_). Overall control performance is affected by the accuracy of these parameters. The results showed that our controller was able to alleviate the challenges due to poorly adjusted *θ*_treatment_ to some extent as explored in Scenario B. Yet, the best performance occurred when *θ*_treatment_ was properly adjusted for pregnancy.

Since the UVA/Padova simulator does not contain a validated pregnancy cohort, and the baseline zone-MPC is a controller that is already clinically validated, we use the 10-subject version of the simulator^3^ for evaluating the impact of our tuning to meet the pregnancy-specific control targets. While the use of the same *in-silico* subjects both for tuning and *in-silico* testing purposes is a limitation of our work, the results from our pilot clinical trial, summarized in *Section 4.2*, provide insight on the controller’s performance on a separate testing cohort under real-life conditions.

Finally, our current approach of exhaustive search for *ψ* is amenable to an optimization-guided procedure using global optimization methods such as Bayesian optimization (44). Thus, we can automate the search for *ψ* and *θ*_treatment_ such that they are partially tuned *offline* (using historical data) and partially tuned *online* (using patient’s real-time data). Of note, this automated alternative would require a multi-objective approach where different weights, typically manually tuned, are assigned on different glycemic metrics and on the outcomes of each scenario.

### 4.4 Conclusions

We presented a zone-MPC controller tuned for pregnancy complicated with T1D. Through extensive simulation scenarios, the pregnancy-specific zone-MPC improved performance in the pregnancy target range when compared to the baseline zone-MPC. Our findings emphasize the need for customized glucose control systems for pregnancy.

Our work provides the first *in-silico* results for CLC performance in T1D pregnancy and can be employed as a baseline for *in-silico* evaluation of future efforts in this area. This example of tailored design also provides a strategy for developing and verifying tailored solutions to target specific sub-populations with T1D, which is a mostly unmet need (7). As the use of CLC systems becomes more prevalent, understanding how these systems can be customized to the needs of specific sub-populations will be critical to extend the reach and ensuring satisfactory performance. We believe that the progress toward providing closed-loop glucose control option for all individuals with T1D will be accelerated greatly by algorithms with flexible components that can be tuned for differing physiological challenges and treatment requirements.

## Data Availability Statement

The original contributions presented in the study are included in the article/**Supplementary Material**. Further inquiries can be directed to the corresponding author.

## Author Contributions

BO: Conceptualization, methodology, software, validation, formal analysis, investigation, resources, data curation, writing—original draft, writing—review and editing, visualization. SD: Methodology, software, validation, resources, writing—review and editing. FJD: Resources, writing— review and editing, supervision. ED: Conceptualization, methodology, validation, investigation, resources, writing—review and editing, supervision, project administration, funding acquisition. All authors contributed to the article and approved the submitted version.

## Funding

National Institutes of Health (R01DK120358).

## Conflict of Interest

FJD reports equity, licensed IP and is a member of the Scientific Advisory Board of Mode AGC. ED reports receiving grants from JDRF, NIH, and Helmsley Charitable Trust, personal fees from Roche and Eli Lilly, patents on artificial pancreas technology, and product support from Dexcom, Insulet, Tandem, and Roche. ED is currently an employee and shareholder of Eli Lilly and Company. The work presented in this manuscript was performed as part of his academic appointment and is independent of his employment with Eli Lilly and Company.

The remaining authors declare that the research was conducted. In the absence of any commercial or financial relationships that could be construed as a potential conflict of interest.

The handling editor declared a past co-authorship with one of the authors BO within the past two years.

## Publisher’s Note

All claims expressed in this article are solely those of the authors and do not necessarily represent those of their affiliated organizations, or those of the publisher, the editors and the reviewers. Any product that may be evaluated in this article, or claim that may be made by its manufacturer, is not guaranteed or endorsed by the publisher.

## Footnotes

1 Tandem Diabetes Care Announces Expanded Pediatric Indication of the t:slim X2 Insulin Pump withControl-IQ Advanced Hybrid Closed-Loop Technology, available in https://bit.ly/3qyy1JA.

2 FDA approves automated insulin delivery and monitoring system for use in younger pediatric patients, available in https://bit.ly/3jwuAlm.

3 Distributed Version of the UVA/Padova simulator (https://tegvirginia.com/software/t1dms/) contains a smaller sample of *in-silico* subjects for developing and testing treatment strategies.
