# Supplementary material for: Zone-MPC Automated Insulin Delivery Algorithm Tuned for Pregnancy Complicated by Type 1 Diabetes
Source: Front Endocrinol (Lausanne). 2022 Mar 22;12:768639. doi: 10.3389/fendo.2021.768639 (PMC8982146; doi:10.3389/fendo.2021.768639)
Supplement: Supplementary file 1 [file Presentation_1.pdf]

Supplementary Material

1 Summary plots

In this section, we provide average glucose trajectories and insulin inputs for baseline and pregnancy-specific zone-MPC and under each scenario.

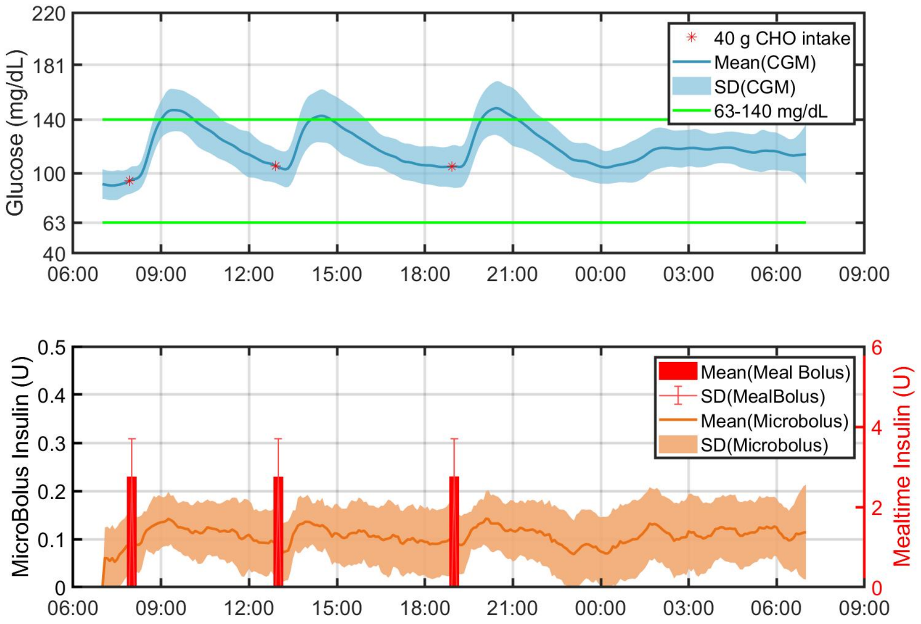

(a) Baseline Controller Design: Treatment parameters are tuned for pregnancy and meal boluses are administered at mealtimes

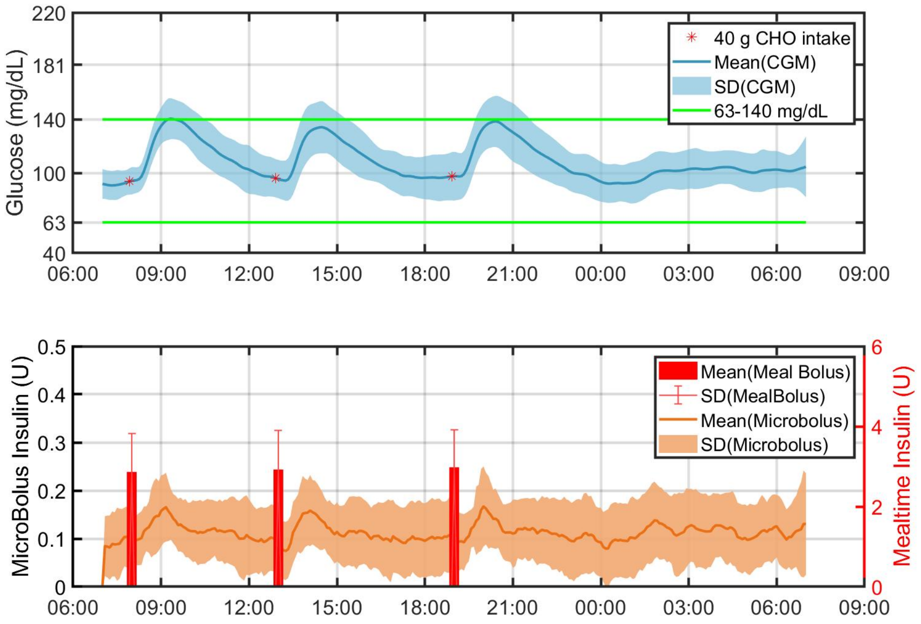

(b) Pregnancy-specific Controller Design: Treatment parameters are tuned for pregnancy and meal boluses are administered at mealtimes

Figure 1: Summary plots of insulin injection and glucose outcomes under Scenario A.1

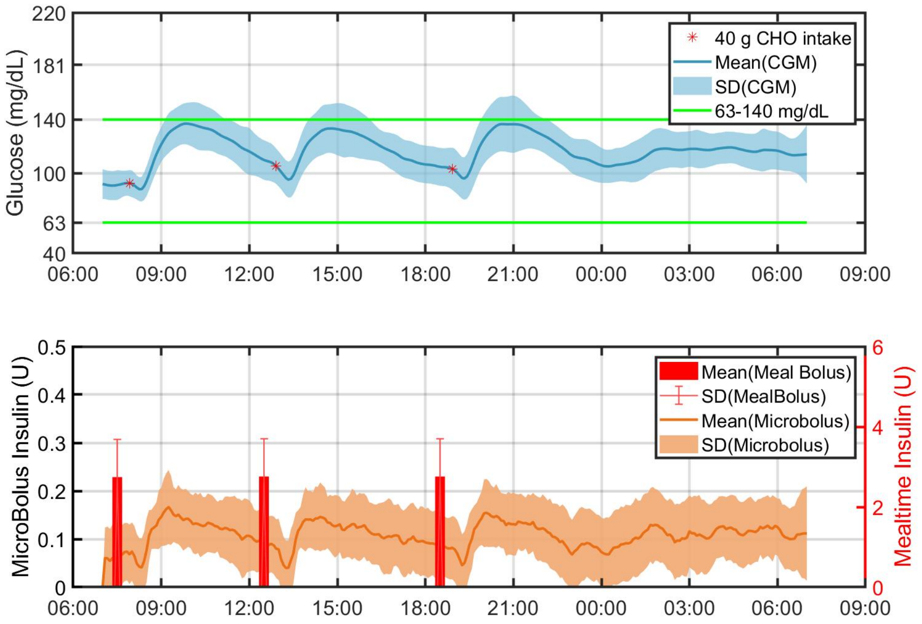

(a) Baseline Controller Design: Treatment parameters are tuned for pregnancy and meal boluses are administered 30 minutes before mealtime

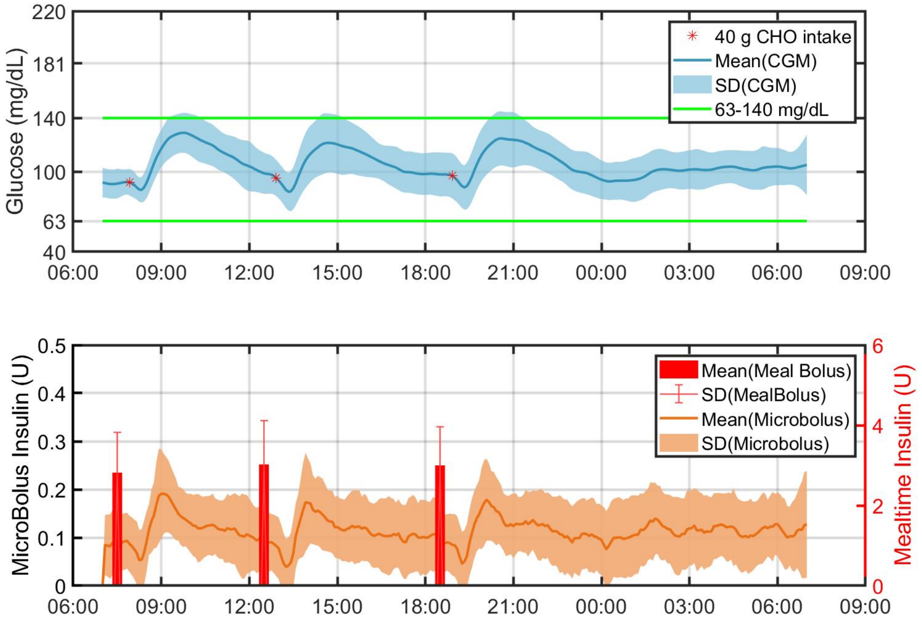

(b) Pregnancy-specific Controller Design: Treatment parameters are tuned for pregnancy and meal boluses are administered 30 minutes before mealtime

Figure 2: Summary plots of insulin injection and glucose outcomes under Scenario A.2

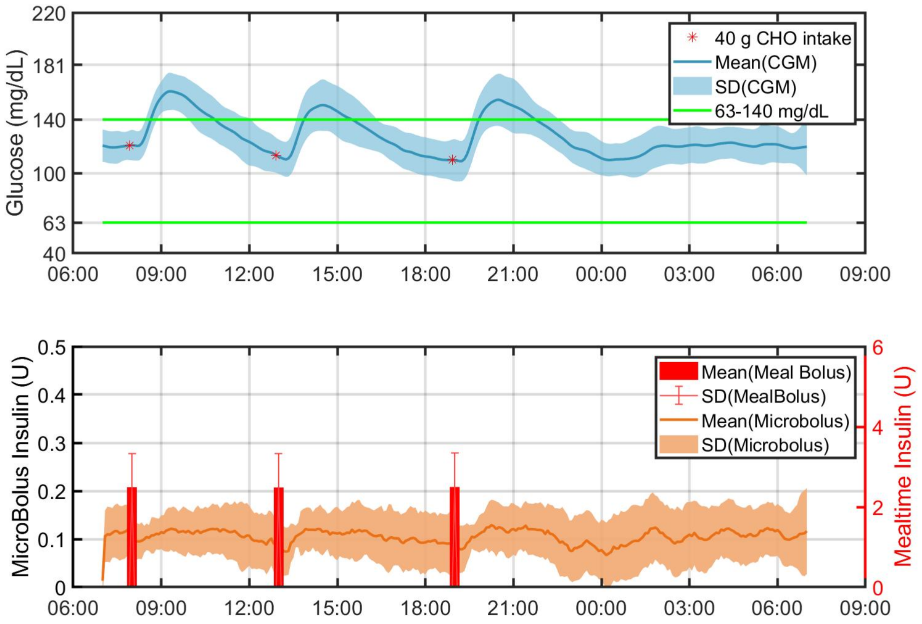

(a) Baseline Controller Design: Treatment parameters are not tuned for pregnancy and meal boluses are administered at mealtimes

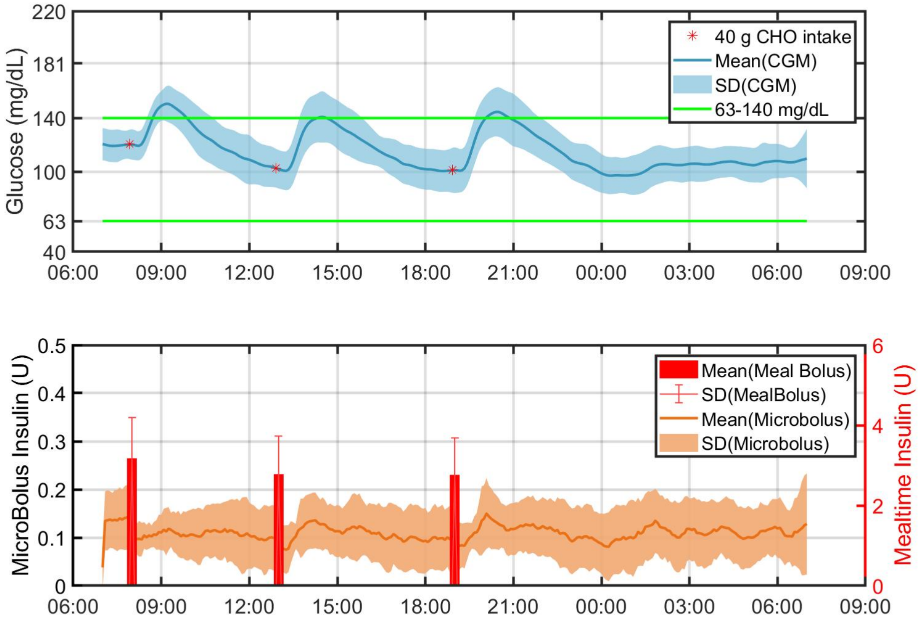

(b) Pregnancy-specific Controller Design: Treatment parameters are not tuned for pregnancy and meal boluses are administered at mealtimes

Figure 3: Summary plots of insulin injection and glucose outcomes under Scenario B.1a

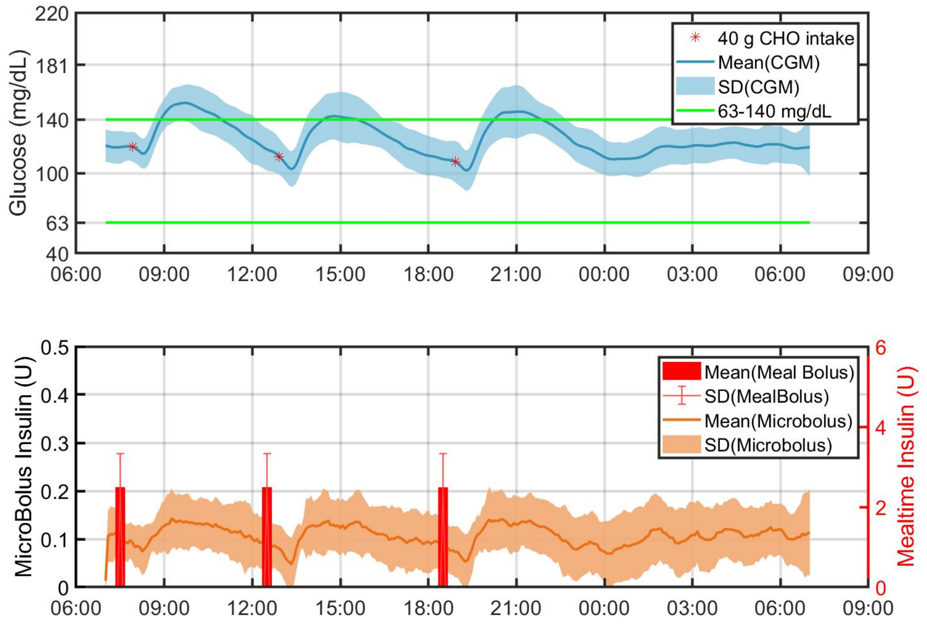

(a) Baseline Controller Design: Treatment parameters are not tuned for pregnancy and meal boluses are administered 30 minutes before mealtime

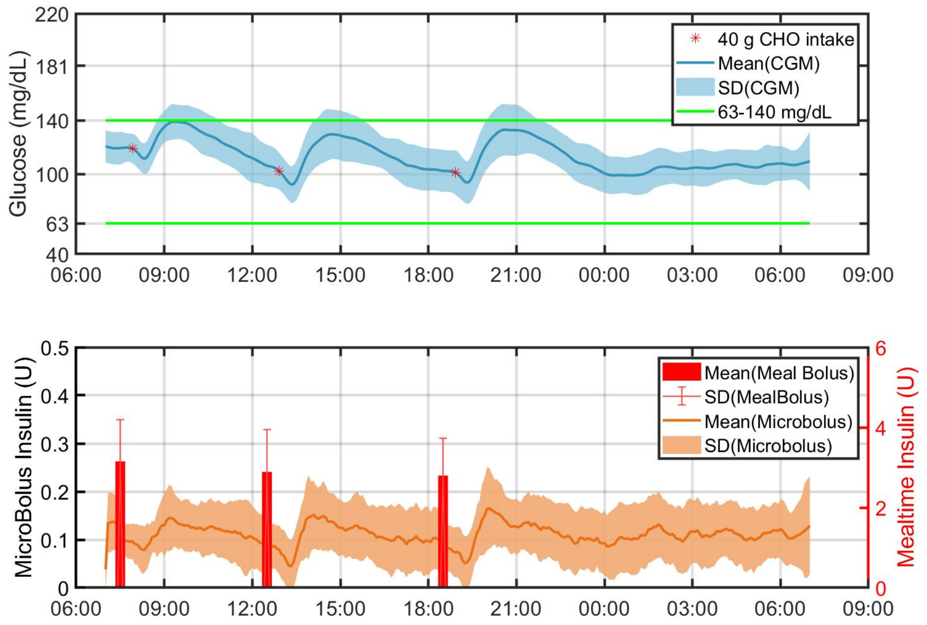

(b) Pregnancy-specific Controller Design: Treatment parameters are not tuned for pregnancy and meal boluses are administered 30 minutes before mealtime

Figure 4: Summary plots of insulin injection and glucose outcomes under Scenario B.1b

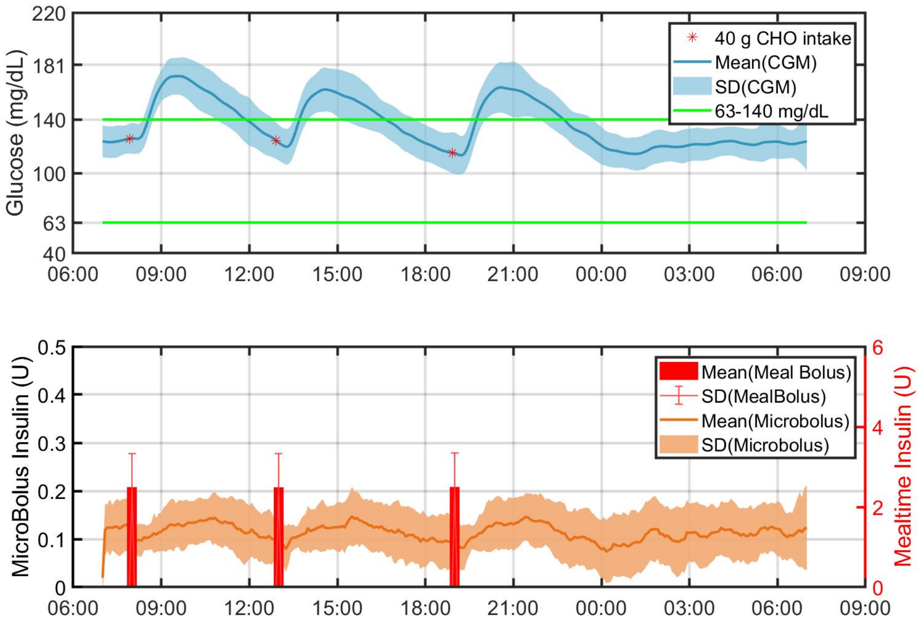

(a) Baseline Controller Design: Treatment parameters not tuned for pregnancy, subjects are 25% more insulin resistant, and meal boluses are administered at mealtimes

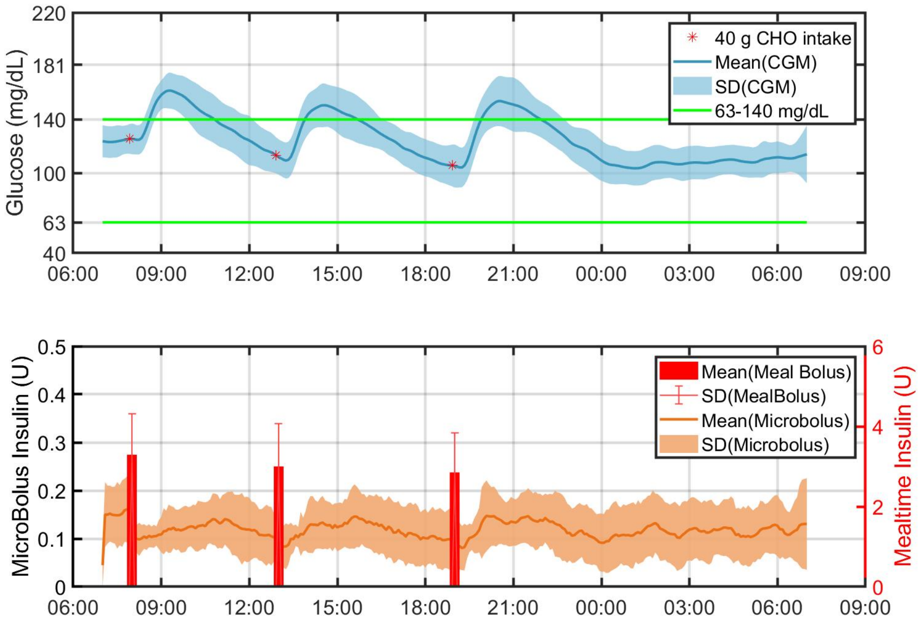

(b) Pregnancy-specific Controller Design: Treatment parameters not tuned for pregnancy, subjects are 25% more insulin resistant, and meal boluses are administered at mealtimes

Figure 5: Summary plots of insulin injection and glucose outcomes under Scenario B.2a

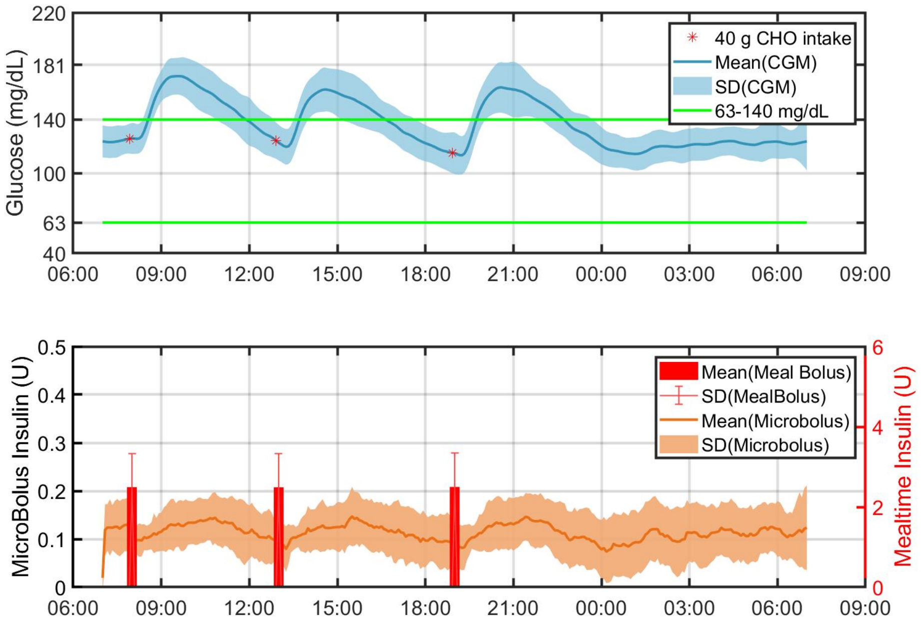

(a) Baseline Controller Design: Treatment parameters are tuned for pregnancy, subjects are 25% more insulin resistant, and meal boluses are given 30 minutes before mealtime

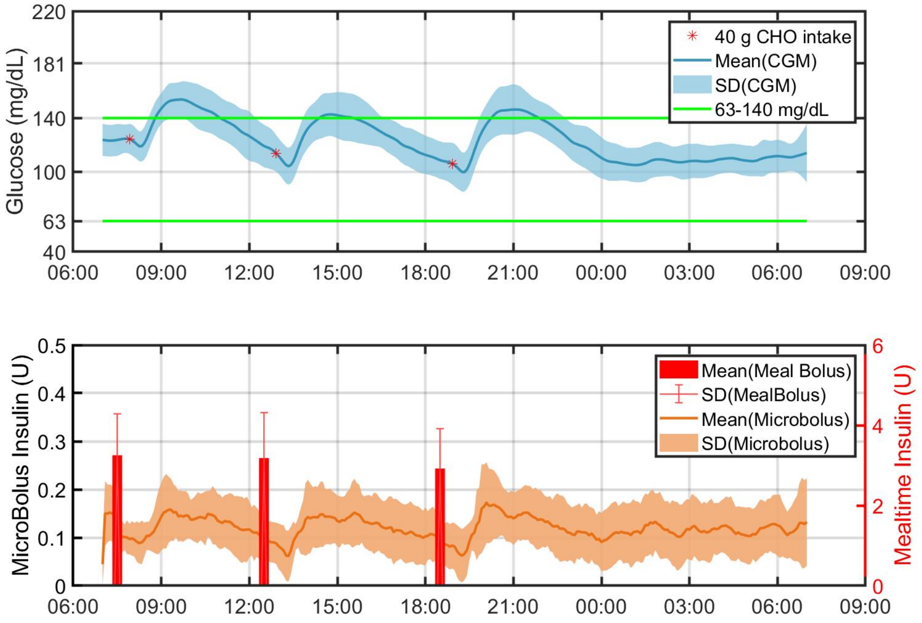

(b) Pregnancy-specific Controller Design: Treatment parameters are not tuned for pregnancy, subjects are 25% more insulin resistant, and meal boluses are given 30 minutes before mealtime

Figure 6: Summary plots of insulin injection and glucose outcomes under Scenario B.2b

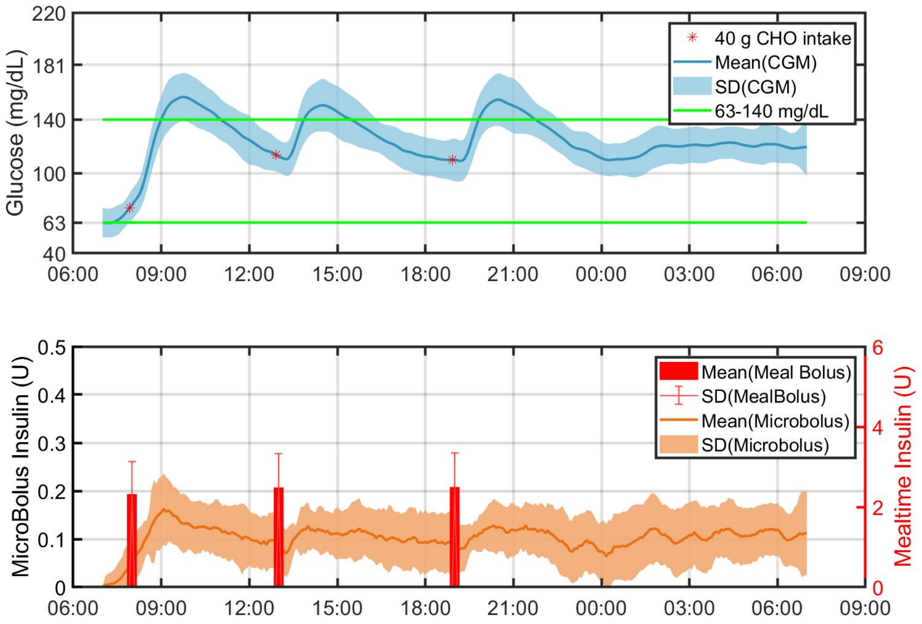

(a) Baseline Controller Design: Treatment parameters are not tuned for pregnancy, initial glucose is 60 mg/dL, and meal boluses are administered at mealtimes

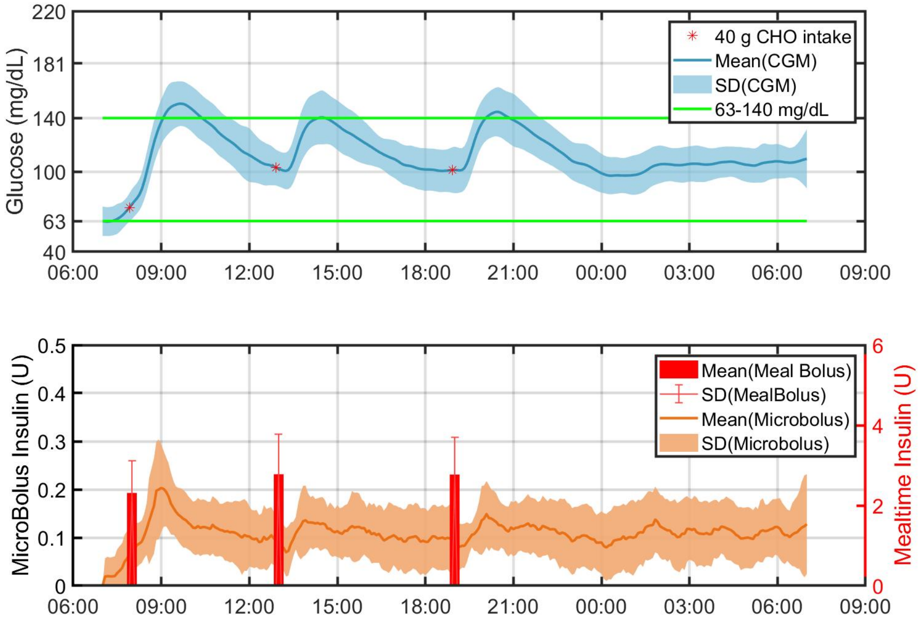

(b) Pregnancy-specific Controller Design: Treatment parameters are not tuned for pregnancy, initial glucose is 60 mg/dL, and meal boluses are administered at mealtimes

Figure 7: Summary plots of insulin injection and glucose outcomes under Scenario B.3a

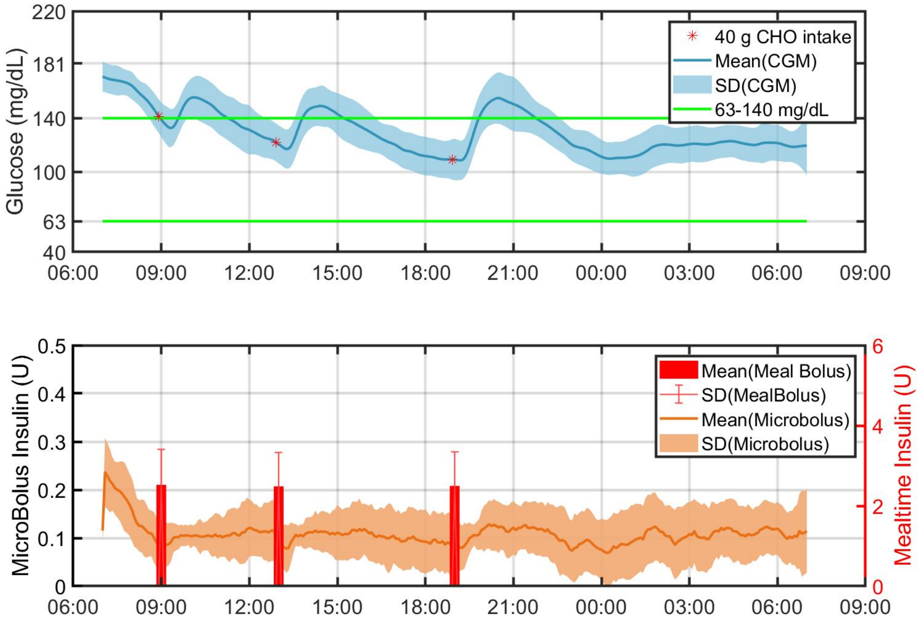

(a) Baseline Controller Design: Treatment parameters are not tuned for pregnancy, initial glucose is 170 mg/dL, and meal boluses are administered at mealtimes

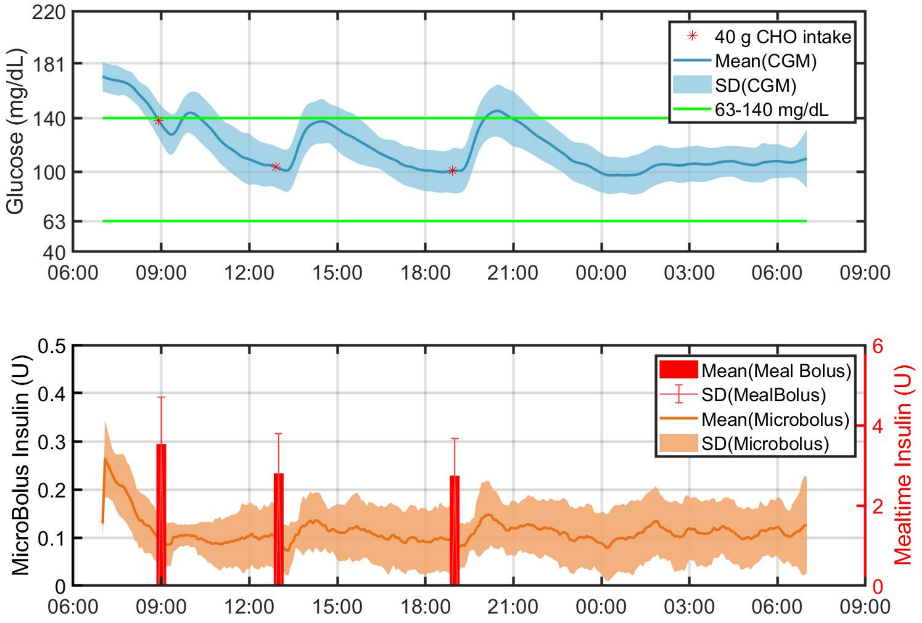

(b) Pregnancy-specific Controller Design: Treatment parameters are not tuned for pregnancy, initial glucose is 170 mg/dL, and meal boluses are administered at mealtimes

Figure 8: Summary plots of insulin injection and glucose outcomes under Scenario B.3b

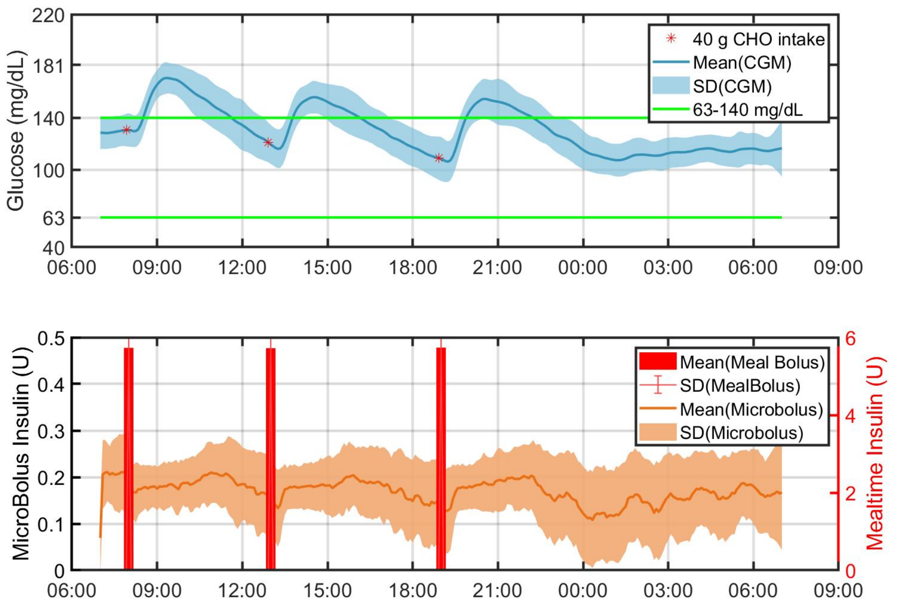

(a) Baseline Controller Design: High Insulin Resistance with Treatment Regimen Adjusted for Pregnancy. Meal boluses are administered at mealtimes

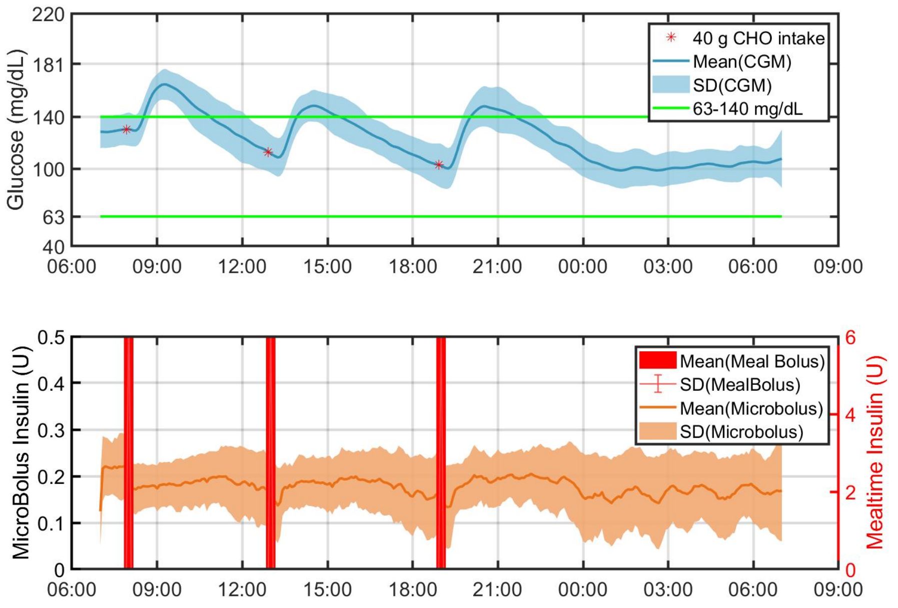

(b) Pregnancy-specific Controller Design: High Insulin Resistance with Treatment Regimen Adjusted for Pregnancy. Meal boluses are administered at mealtimes

Figure 9: Summary plots of insulin injection and glucose outcomes under Scenario C.1

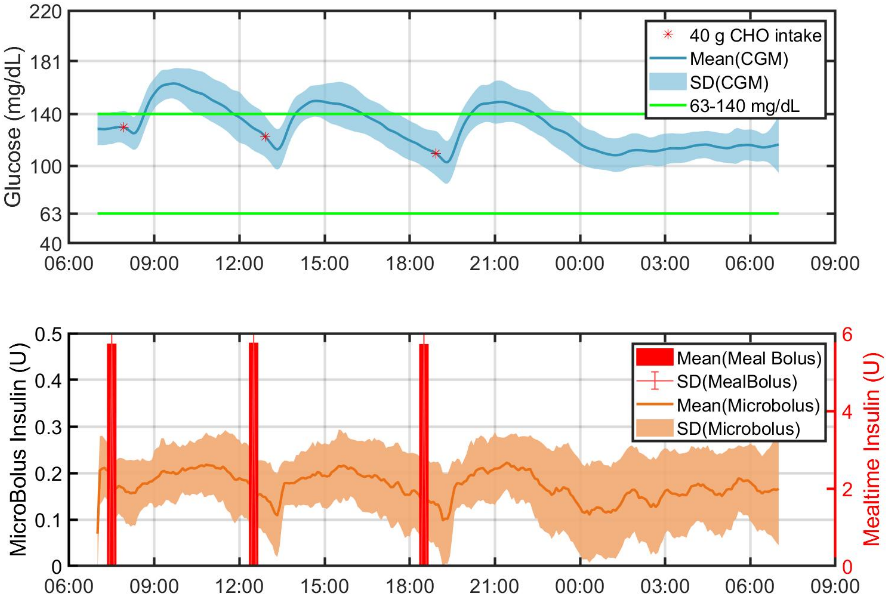

(a) Baseline Controller Design: High Insulin Resistance with Treatment Regimen Adjusted for Pregnancy. Meal boluses are administered 30 minutes before mealtimes

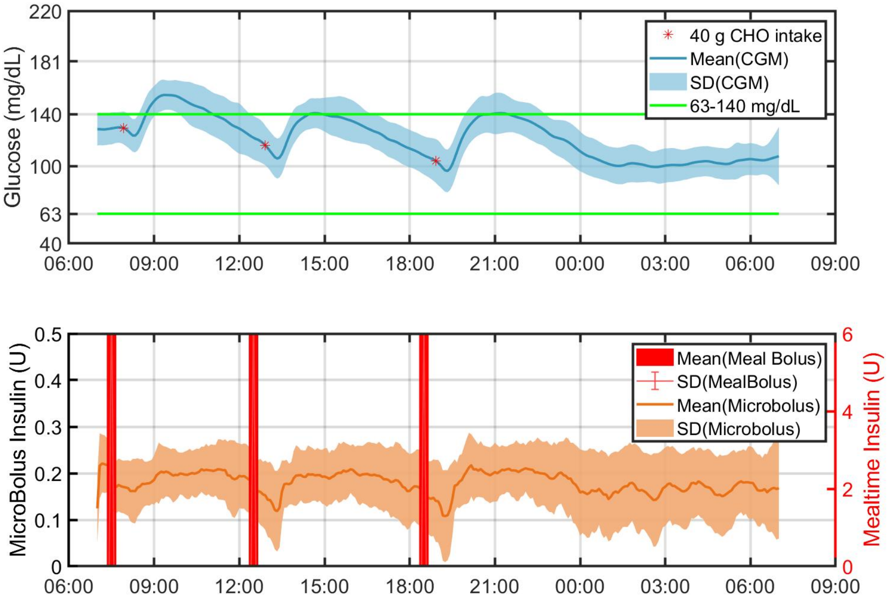

(b) Pregnancy-specific Controller Design:High Insulin Resistance with Treatment Regimen Adjusted for Pregnancy. Meal boluses are administered 30 minutes before mealtimes

Figure 10: Summary plots of insulin injection and glucose outcomes under Scenario C.2

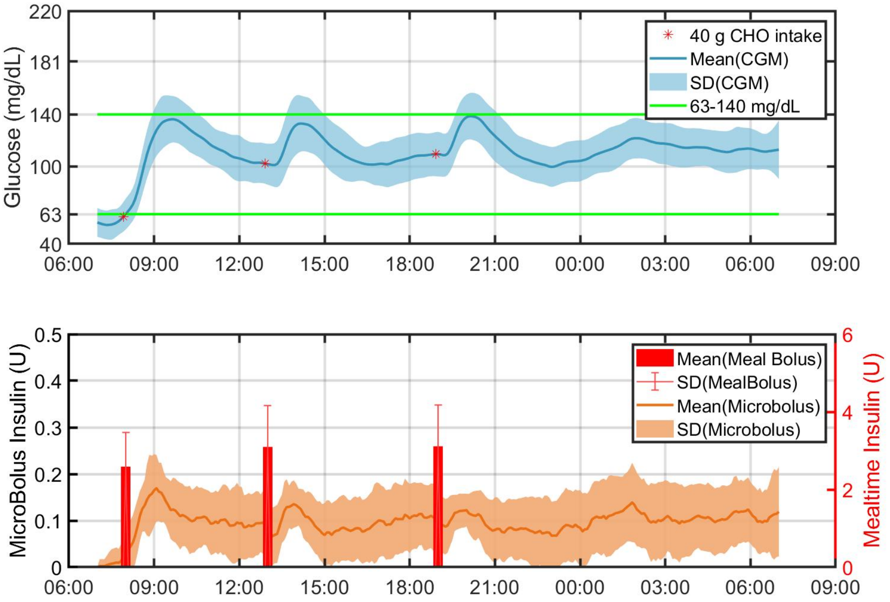

(a) Baseline Controller Design: Hypoglycemia prone extreme-case scenario

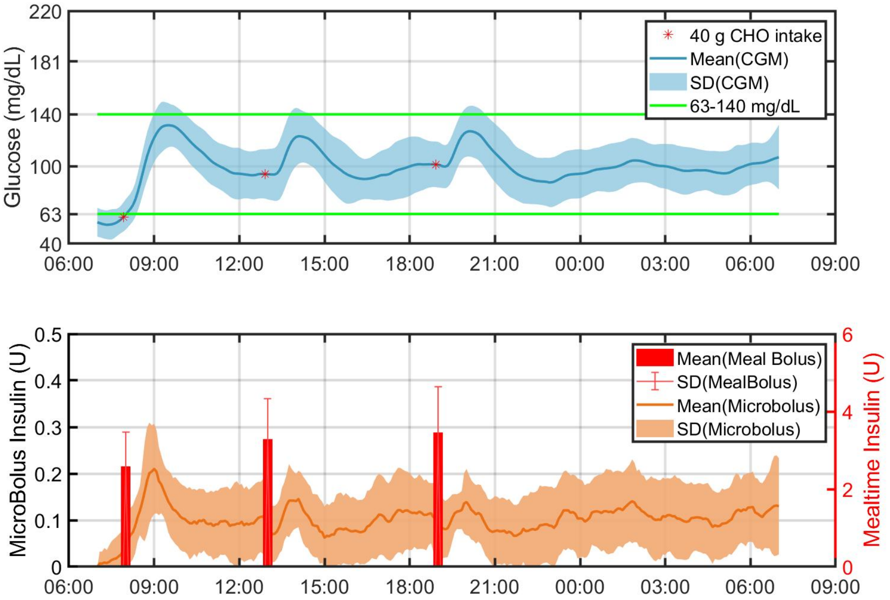

(b) Pregnancy-specific Controller Design: Hypoglycemia prone extreme-case scenario

Figure 11: Summary plots of insulin injection and glucose outcomes under Scenario D.1

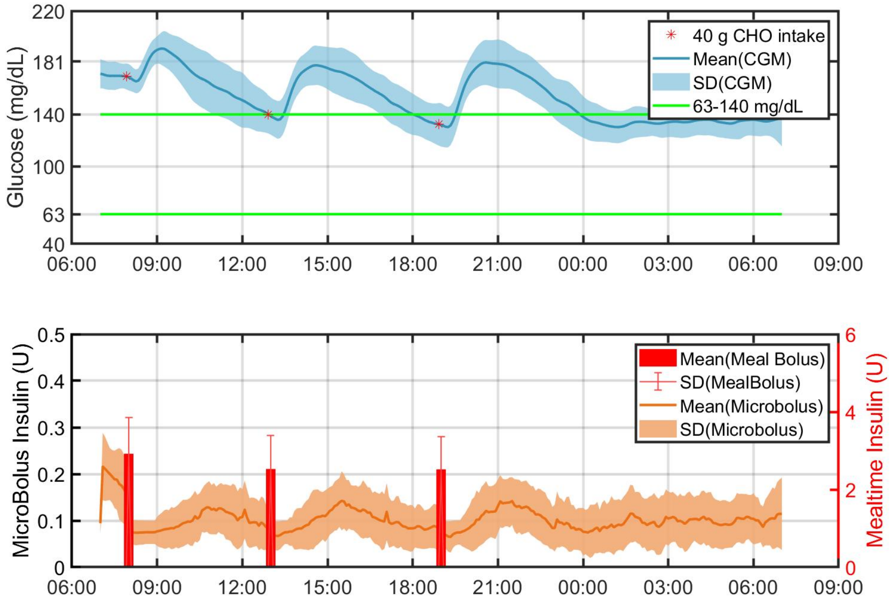

(a) Baseline Controller Design: Hyperglycemia prone extreme-case scenario

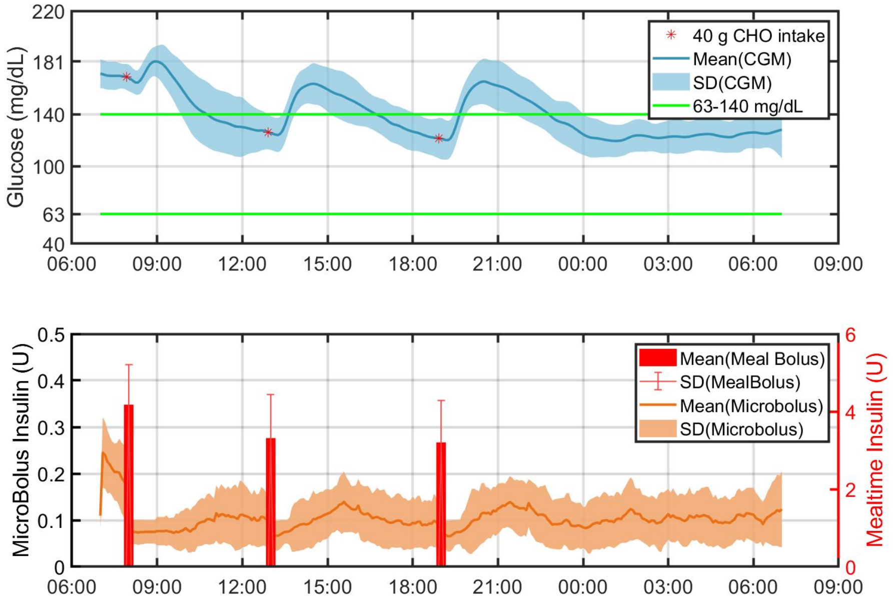

(b) Pregnancy-specific Controller Design: Hyperglycemia prone extreme-case scenario

Figure 12: Summary plots of insulin injection and glucose outcomes under Scenario D.2

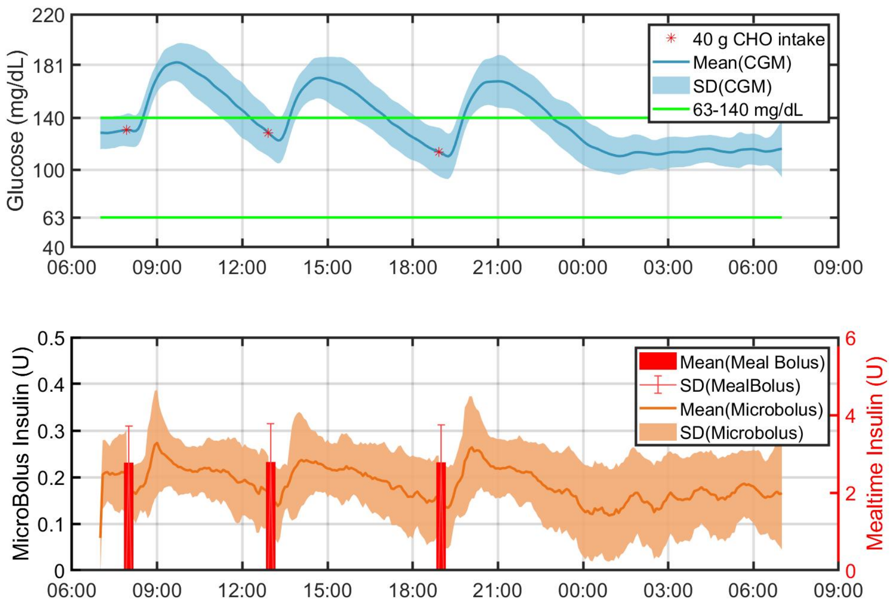

(a) Baseline Controller Design: High insulin resistance with poorly adjusted CR, extreme-case scenario

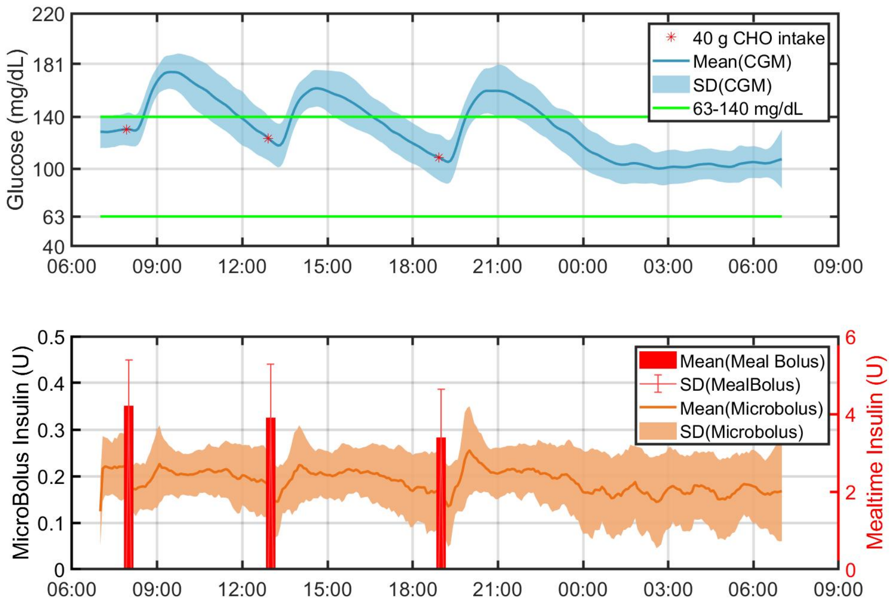

(b) Pregnancy-specific Controller Design: High insulin resistance with poorly adjusted CR, extreme-case scenario

Figure 13: Summary plots of insulin injection and glucose outcomes under Scenario D.3

2 IOB Curves

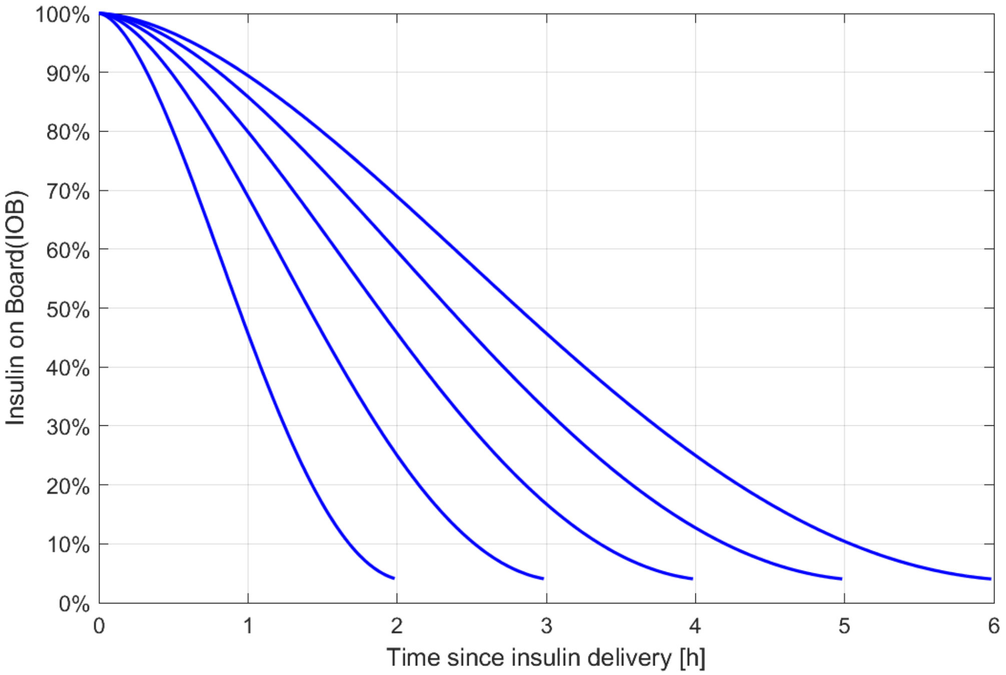

Figure 14: Exemplary IOB decay curves of length 2 hours to 6 hours

3 P-values from paired t-tests

Here we provide p-values obtained for the differences between the zone-adjusted vs. pregnancy-specific controller performances presented in Table 3 and 4.

Table 1: P-values for zone-adjusted vs. pregnancy controller performance evaluated for pregnancy glycemic targets

Scenario	% Time < 54 mg/dL	% Time < 63 mg/dL	% Time 63-140 mg/dL	% Time > 140 mg/dL
A.1	0.140	0.019	0.135	0.039
A.2	0.161	0.056	0.119	0.074
B.1.a	0.274	0.069	0.000	0.000
B.1.b	0.343	0.051	0.000	0.000
B.2.a	NaN	0.343	0.000	0.000
B.2.b	0.343	0.620	0.000	0.000
B.3.a	0.186	0.045	0.000	0.000
B.3.b	0.197	0.058	0.001	0.000
C.1	0.343	0.232	0.001	0.001
C.2	0.343	0.419	0.000	0.000
D.1	0.016	0.005	0.019	0.133
D.2	NaN	0.343	0.000	0.000
D.3	0.343	0.343	0.000	0.000

Table 2: P-values for zone-adjusted vs. pregnancy controller performance evaluated for standard glycemic targets

Scenario	% Time < 70 mg/dL	% Time 70-180 mg/dL	% Time > 180 mg/dL	% Time > 250 mg/dL
A.1	0.018	0.114	0.342	NaN
A.2	0.010	0.015	0.320	NaN
B.1.a	0.042	0.939	0.206	NaN
B.1.b	0.048	0.843	0.252	NaN
B.2.a	0.237	0.046	0.043	NaN
B.2.b	0.180	0.066	0.062	NaN
B.3.a	0.030	0.961	0.261	NaN
B.3.b	0.029	0.292	0.118	NaN
C.1	0.897	0.000	0.016	NaN
C.2	0.275	0.040	0.025	NaN
D.1	0.001	0.001	0.343	NaN
D.2	0.343	0.001	0.001	0.343
D.3	0.509	0.011	0.011	NaN

4 Post-hoc Evaluation of Parameter Choices

Next four pages show results of additional exemplary analyses that explore local sensitivity of metrics around ψ_p under Scenarios A.2 and B.1.a for selected pairs of elements in ψ.

Analysis I: IOB decay curve length (λ) and threshold for an additional correction bolus along with the meal bolus threshold (τ_c)

Scenario A.2

63-140 mg/dL

λ \ τ_c	90	100	110	120	130
1	96.56%	96.45%	96.41%	96.41%	96.41%
2	96.66%	96.47%	96.32%	96.35%	96.35%
3	96.00%	95.98%	95.51%	95.54%	95.54%
4	94.80%	94.52%	94.48%	94.44%	94.44%
5	95.27%	94.61%	94.60%	94.68%	94.68%
6	94.87%	94.25%	93.91%	93.71%	93.71%

< 63 mg/dL

λ \ τ_c	90	100	110	120	130
1	0.10%	0.10%	0.10%	0.10%	0.10%
2	0.11%	0.10%	0.10%	0.10%	0.10%
3	0.21%	0.19%	0.19%	0.19%	0.19%
4	0.36%	0.51%	0.51%	0.51%	0.51%
5	0.36%	0.07%	0.07%	0.07%	0.07%
6	0.36%	0.14%	0.14%	0.14%	0.14%

> 140 mg/dL

λ \ τ_c	90	100	110	120	130
1	3.3%	3.4%	3.5%	3.5%	3.5%
2	3.2%	3.4%	3.6%	3.6%	3.6%
3	3.8%	3.8%	4.3%	4.3%	4.3%
4	4.8%	5.0%	5.0%	5.1%	5.1%
5	4.5%	5.3%	5.3%	5.3%	5.3%
6	5.0%	5.6%	5.9%	6.2%	6.2%

Scenario B.1.a

63-140 mg/dL

λ \ τ_c	90	100	110	120	130
1	89.50%	89.36%	88.81%	88.78%	88.29%
2	88.53%	87.91%	87.32%	87.18%	86.97%
3	87.68%	87.08%	85.77%	85.61%	85.35%
4	87.23%	87.05%	86.38%	85.90%	85.50%
5	86.91%	86.76%	86.04%	85.43%	84.77%
6	86.88%	86.77%	85.28%	84.86%	83.91%

< 63 mg/dL

λ \ τ_c	90	100	110	120	130
1	0.00%	0.00%	0.00%	0.00%	0.06%
2	0.00%	0.00%	0.00%	0.00%	0.00%
3	0.00%	0.00%	0.00%	0.00%	0.00%
4	0.00%	0.00%	0.00%	0.00%	0.00%
5	0.00%	0.00%	0.00%	0.00%	0.00%
6	0.00%	0.00%	0.00%	0.00%	0.00%

> 140 mg/dL

λ \ τ_c	90	100	110	120	130
1	10.50%	10.64%	11.19%	11.22%	11.65%
2	11.47%	12.09%	12.68%	12.82%	13.03%
3	12.32%	12.92%	14.23%	14.39%	14.65%
4	12.77%	12.95%	13.62%	14.10%	14.50%
5	13.09%	13.24%	13.96%	14.57%	15.23%
6	13.12%	13.23%	14.72%	15.14%	16.09%

Analysis II: The glucose range (G_-^v and G_+^v) where there is a cost associated with glucose velocity

Scenario A.2

63-140 mg/dL

G_-^v \ G_+^v	110	120	130	140	150	160	170	180	190	200	210	220
**110**	95.15%	95.10%	95.14%	95.29%	95.37%	95.88%	95.79%	95.79%	95.79%	95.79%	95.79%	95.79%
**120**	NA	95.15%	95.11%	95.10%	95.39%	95.63%	95.98%	95.98%	95.98%	95.98%	95.98%	95.98%
**130**	NA	NA	95.15%	95.11%	95.26%	95.47%	95.80%	95.80%	95.80%	95.80%	95.80%	95.80%
**140**	NA	NA	NA	95.15%	94.80%	95.19%	95.23%	95.23%	95.23%	95.23%	95.23%	95.23%

< 63 mg/dL

G_-^v \ G_+^v	110	120	130	140	150	160	170	180	190	200	210	220
**110**	0.06%	0.04%	0.07%	0.24%	0.22%	0.22%	0.22%	0.22%	0.22%	0.22%	0.22%	0.22%
**120**	NA	0.06%	0.00%	0.11%	0.19%	0.19%	0.19%	0.19%	0.19%	0.19%	0.19%	0.19%
**130**	NA	NA	0.06%	0.03%	0.20%	0.20%	0.20%	0.20%	0.20%	0.20%	0.20%	0.20%
**140**	NA	NA	NA	0.06%	0.15%	0.26%	0.26%	0.26%	0.26%	0.26%	0.26%	0.26%

> 140 mg/dL

G_-^v \ G_+^v	110	120	130	140	150	160	170	180	190	200	210	220
**110**	4.80%	4.86%	4.80%	4.47%	4.41%	3.90%	3.99%	3.99%	3.99%	3.99%	3.99%	3.99%
**120**	NA	4.80%	4.89%	4.79%	4.42%	4.18%	3.83%	3.83%	3.83%	3.83%	3.83%	3.83%
**130**	NA	NA	4.80%	4.86%	4.54%	4.33%	4.00%	4.00%	4.00%	4.00%	4.00%	4.00%
**140**	NA	NA	NA	4.80%	5.05%	4.55%	4.52%	4.52%	4.52%	4.52%	4.52%	4.52%

Scenario B.1.a

63-140 mg/dL

G_-^v \ G_+^v	110	120	130	140	150	160	170	180	190	200	210	220
**110**	95.15%	95.10%	95.14%	95.29%	95.37%	95.88%	95.79%	95.79%	95.79%	95.79%	95.79%	95.79%
**120**	NA	95.15%	95.11%	95.10%	95.39%	95.63%	95.98%	95.98%	95.98%	95.98%	95.98%	95.98%
**130**	NA	NA	95.15%	95.11%	95.26%	95.47%	95.80%	95.80%	95.80%	95.80%	95.80%	95.80%
**140**	NA	NA	NA	95.15%	94.80%	95.19%	95.23%	95.23%	95.23%	95.23%	95.23%	95.23%

< 63 mg/dL

G_-^v \ G_+^v	110	120	130	140	150	160	170	180	190	200	210	220
**110**	0.06%	0.04%	0.07%	0.24%	0.22%	0.22%	0.22%	0.22%	0.22%	0.22%	0.22%	0.22%
**120**	NA	0.06%	0.00%	0.11%	0.19%	0.19%	0.19%	0.19%	0.19%	0.19%	0.19%	0.19%
**130**	NA	NA	0.06%	0.03%	0.20%	0.20%	0.20%	0.20%	0.20%	0.20%	0.20%	0.20%
**140**	NA	NA	NA	0.06%	0.15%	0.26%	0.26%	0.26%	0.26%	0.26%	0.26%	0.26%

> 140 mg/dL

G_-^v \ G_+^v	110	120	130	140	150	160	170	180	190	200	210	220
**110**	4.80%	4.86%	4.80%	4.47%	4.41%	3.90%	3.99%	3.99%	3.99%	3.99%	3.99%	3.99%
**120**	NA	4.80%	4.89%	4.79%	4.42%	4.18%	3.83%	3.83%	3.83%	3.83%	3.83%	3.83%
**130**	NA	NA	4.80%	4.86%	4.54%	4.33%	4.00%	4.00%	4.00%	4.00%	4.00%	4.00%
**140**	NA	NA	NA	4.80%	5.05%	4.55%	4.52%	4.52%	4.52%	4.52%	4.52%	4.52%

Note: Results for $G_+^v \geq 170$ mg/dL are the same across columns because the controller manages to keep glucose below 180 mg/dL throughout.

Analysis III: Reference glucose (G_{ref}) and bolus reduction threshold (G^m)

Scenario A.2

63-140 mg/dL

G_{ref} \ G^m	60	70	80	90	100	110
70	96.64%	96.30%	96.30%	94.98%	94.17%	94.28%
80	96.18%	96.07%	96.18%	94.95%	94.00%	93.60%
90	96.25%	95.98%	95.98%	94.46%	93.70%	93.99%
100	95.68%	95.42%	95.63%	94.54%	93.25%	92.59%
110	95.33%	94.96%	94.80%	93.70%	92.02%	91.97%

< 63 mg/dL

G_{ref} \ G^m	60	70	80	90	100	110
70	0.00%	0.10%	0.10%	0.08%	0.08%	0.08%
80	0.35%	0.24%	0.13%	0.08%	0.12%	0.12%
90	0.19%	0.19%	0.19%	0.03%	0.07%	0.07%
100	0.37%	0.37%	0.16%	0.02%	0.01%	0.01%
110	0.03%	0.03%	0.03%	0.03%	0.00%	0.00%

> 140 mg/dL

G_{ref} \ G^m	60	70	80	90	100	110
70	3.36%	3.60%	3.60%	4.95%	5.75%	5.63%
80	3.46%	3.69%	3.69%	4.97%	5.88%	6.27%
90	3.57%	3.83%	3.83%	5.51%	6.23%	5.94%
100	3.96%	4.21%	4.21%	5.44%	6.75%	7.40%
110	4.64%	5.01%	5.17%	6.27%	7.98%	8.03%

Scenario B.1.a

63-140 mg/dL

G_{ref} \ G^m	60	70	80	90	100	110
70	88.58%	88.58%	88.47%	87.70%	86.59%	85.70%
80	87.55%	87.55%	87.34%	86.93%	85.37%	84.77%
90	87.08%	87.08%	87.00%	86.71%	85.18%	83.84%
100	86.41%	86.41%	86.23%	86.36%	84.61%	83.27%
110	86.04%	86.04%	85.93%	85.52%	84.31%	82.59%

< 63 mg/dL

G_{ref} \ G^m	60	70	80	90	100	110
70	0.03%	0.03%	0.03%	0.00%	0.00%	0.00%
80	0.00%	0.00%	0.00%	0.00%	0.00%	0.00%
90	0.00%	0.00%	0.00%	0.00%	0.00%	0.00%
100	0.00%	0.00%	0.00%	0.00%	0.00%	0.00%
110	0.00%	0.00%	0.00%	0.00%	0.00%	0.00%

> 140 mg/dL

G_{ref} \ G^m	60	70	80	90	100	110
70	11.38%	11.38%	11.49%	12.30%	13.41%	14.30%
80	12.45%	12.45%	12.66%	13.07%	14.63%	15.23%
90	12.92%	12.92%	13.00%	13.29%	14.82%	16.16%
100	13.59%	13.59%	13.77%	13.64%	15.39%	16.73%
110	13.96%	13.96%	14.07%	14.48%	15.69%	17.41%

The value selected for ψ_p

Poorest performance — Highest performance*

*Color codes are applied separately for each glycemic metric and are meant to help the evaluation of performances across parameter selections within each scenario.
